# Effects of sedentary behaviour interventions on biomarkers of cardiometabolic risk in adults: systematic review with meta-analyses

**DOI:** 10.1136/bjsports-2019-101154

**Published:** 2020-04-08

**Authors:** Nyssa T Hadgraft, Elisabeth Winkler, Rachel E Climie, Megan S Grace, Lorena Romero, Neville Owen, David Dunstan, Genevieve Healy, Paddy C Dempsey

**Affiliations:** 1 Centre for Urban Transitions, Swinburne University of Technology, Melbourne, VIC, Australia; 2 Baker Heart and Diabetes Institute, Melbourne, VIC, Australia; 3 School of Public Health, The University of Queensland, Brisbane, QLD, Australia; 4 The Alfred Hospital, Melbourne, VIC, Australia; 5 Central Clinical School/Department of Epidemiology and Preventive Medicine, Faculty of Medicine, Nursing and Health Sciences, Monash University, Melbourne, VIC, Australia; 6 Institute of Physical Activity and Nutrition Research, School of Exercise and Nutrition Sciences, Deakin University, Melbourne, VIC, Australia; 7 Mary MacKillop Institute of Health Research, Australian Catholic University, Melbourne, VIC, Australia; 8 School of Sport Science, Exercise and Health, The University of Western Australia, Perth, WA, Australia; 9 School of Physiotherapy and Exercise Science, Curtin University, Perth, WA, Australia; 10 MRC Epidemiology Unit, Institute of Metabolic Science, University of Cambridge, Cambridge Biomedical Campus, Cambridge, UK

**Keywords:** intervention, sedentary, physical activity, meta-analysis, cardiovascular

## Abstract

**Context/purpose:**

Observational and acute laboratory intervention research has shown that excessive sedentary time is associated adversely with cardiometabolic biomarkers. This systematic review with meta-analyses synthesises results from free living interventions targeting reductions in sedentary behaviour alone or combined with increases in physical activity.

**Methods:**

Six electronic databases were searched up to August 2019 for sedentary behaviour interventions in adults lasting for ≥7 days publishing cardiometabolic biomarker outcomes covering body anthropometry, blood pressure, glucose and lipid metabolism, and inflammation (54 studies). The pooled effectiveness of intervention net of control on 15 biomarker outcomes was evaluated using random effects meta-analyses in the studies with control groups not providing other relevant interventions (33 studies; 6–25 interventions analysed).

**Results:**

Interventions between 2 weeks and <6 months in non-clinical populations from North America, Europe and Australia comprised much of the evidence base. Pooled effects revealed small, significant (p<0.05) beneficial effects on weight (≈ −0.6 kg), waist circumference (≈ −0.7 cm), percentage body fat (≈ −0.3 %), systolic blood pressure (≈ −1.1 mm Hg), insulin (≈ −1.4 pM) and high-density lipoprotein cholesterol (≈ 0.04 mM). Pooled effects on the other biomarkers (p>0.05) were also small, and beneficial in direction except for fat-free mass (≈ 0.0 kg). Heterogeneity ranged widely (I^2^=0.0–72.9).

**Conclusions:**

Our review of interventions targeting sedentary behaviour reductions alone, or combined with increases in physical activity, found evidence of effectiveness for improving some cardiometabolic risk biomarkers to a small degree. There was insufficient evidence to evaluate inflammation or vascular function. Key limitations to the underlying evidence base include a paucity of high-quality studies, interventions lasting for ≥12 months, sensitive biomarkers and clinical study populations (eg, type 2 diabetes).

**PROSPERO trial registration number:**

CRD42016041742

## Introduction

Globally, cardiovascular diseases are the leading cause of death and a major cause of disability and lost productivity in adults.^1 2^ In addition, estimates from 2017 indicate that 451 million people are living with diabetes: a figure projected to rise to 693 million (≈10% of the population) by 2045.^3^

The evidence tends to indicate that greater time spent in sedentary behaviour (ie, sitting/reclining at <1.5 metabolic equivalents (MET))^4^ is adversely associated with the risk of cardiovascular disease, type 2 diabetes and some cancers,^5 6^ and with levels of a range of cardiometabolic risk biomarkers.^7 8^ A less prolonged sedentary accumulation pattern (ie, more regular breaks, shorter sedentary bouts) has also been associated with lower body mass index (BMI).^8^ It has largely been acute laboratory interventions (<7 days) using structured protocols providing experimental evidence that reducing or breaking up sitting can have beneficial effects on certain cardiometabolic biomarkers.^9–12^ For example, compared with uninterrupted sitting time, adding short bouts of light or moderate intensity activity every 20–30 min (generally over a period of 1–5 days) results in improvements to resting blood pressure,^13 14^ fasting and postprandial glucose^15 16^ and insulin,^15 17 18^ and some lipids.^19^

In recognition of the aforementioned evidence, several countries now, in addition to having guidelines concerning physical activity, include guidelines to reduce the quantity of sedentary behaviour and/or break it up.^20–22^ A variety of intervention strategies have been trialled to reduce adults’ levels of sedentary behaviour, particularly in the workplace setting.^23 24^ Reviews indicate these interventions are often effective for reducing sedentary behaviour, especially workplace interventions incorporating environmental modification, ideally as part of a multicomponent intervention.^23 25–27^ What is lacking, however, is an understanding of the nature and extent of health improvements that might be obtained when intervening to reduce sedentary behaviour over longer periods and under free-living conditions. A preliminary evaluation explored this topic (in workplace interventions only) but, having occurred prior to the emergence of several large trials of sedentary behaviour interventions, did not present any meta-analyses and could draw no firm conclusions.^28^

We therefore conducted a systematic review with meta-analyses aiming to synthesise the body of evidence that examined the effectiveness on biomarkers of cardiometabolic risk of ≥7 days interventions that targetted sedentary behaviour (alone or in combination with physical activity) in free-living conditions. We reviewed the evidence on body anthropometry, indicators of blood pressure and related haemodynamics, biomarkers relevant to the metabolism of blood glucose and lipids, and inflammatory biomarkers.

## Methods

This study followed the Preferred Reporting Items for Systematic Reviews and Meta-Analyses (PRISMA)^29^ and the Meta-analysis of Observational Studies in Epidemiology^30^ reporting guidelines.

### Search strategy and study selection

Six electronic databases (Ovid Medline, Ovid Embase, EBM Reviews Cochrane Central, CINAHL, Scopus, Web of Science) were searched systematically from database inception to 27 August 2019 (7 March 2017; 16 February 2018 and, 27 August 2019). A research librarian (LR) conducted an initial search for studies in Medline and Embase and used an analysis of text words and subject terms to develop the search strategies. The final searches were then executed using the appropriate specifications of each database (LR; see online supplementary table S1). Using reference management software (Endnote, Clarivate Analytics, Philadelphia, USA), records were compiled, duplicates were removed, and two authors (NTH and PCD or REC and MSG) performed title and abstract screening and reviewed each full-text article was reviewed against the inclusion criteria. Discrepancies were resolved in consultation with an independent third reviewer (EW).

10.1136/bjsports-2019-101154.supp1 Supplementary data

### Inclusion and exclusion criteria

Inclusion criteria, applied hierarchically, were: (1) reported intervening on sedentary behaviour for ≥7 days; (2) human study; (3) participants all aged ≥18 years; (4) English language; (5) full-length publication; (6) reported as an outcome at least one biomarker of cardiometabolic health, specifically concerning body anthropometry, glucose metabolism, lipid metabolism, blood pressure and related haemodynamics or inflammation (see online supplementary table S2); and, (7) used an intervention study design (single-group preintervention and postintervention, parallel-group design or crossover). To meet criterion (1), the intervention needed to target sedentary behaviour directly or indirectly with replacement of sedentary activity with an alternative (eg, treadmill desks), increasing ‘whole-of-day’ activity (which includes sedentary) or increasing ‘light intensity’ activity (which is almost the inverse of sedentary) or similar. Studies that only mentioned intervening on ‘physical activity’ or exercise could increase these activities at the expense of either sedentary behaviour or light activity, and therefore did not meet criterion (1). Further inclusion criteria for the meta-analyses were: (1) a no-intervention comparison arm (usual care/conditions; attention control) and (2) no other intervention that was likely to provide an appreciable impact on cardiometabolic biomarkers (eg, diet). Physical activity interventions were permitted, since reducing sedentary time very likely increases some form of physical activity as a replacement. Achievement of successful sedentary behavioural change was not considered a requirement for inclusion in the meta-analyses (to avoid potentially overstating effectiveness). Meta-analyses were conducted for each biomarker reported in at least five studies.

10.1136/bjsports-2019-101154.supp2 Supplementary data

### Data extraction

All data were extracted, checked and discrepancies resolved by the review team (NTH, PCD, REC and EW), using standardised rules created a priori. The rules used for regarding extraction, contacting authors for missing or questionable data are in online supplementary table S2. Study quality was assessed using the risk of bias (RoB) V.2.0 tool.^31^

### Statistical analysis

Analyses were performed in STATA V.16 (StataCorp). Significance was set at p<0.05 (two tailed). Pooled effects were estimated based on intervention effects (mean between-groups difference, in units) for the end-of-intervention endpoint extracted from each intervention, with the standard errors multiplied by $\sqrt{(n+1)/2}$ whenever there were n>1 eligible sedentary behaviour intervention arms.^32^ Pooled effects were primarily estimated from random effects (Der Simonian Laird) meta-analysis models, with fixed effects results also reported, along with heterogeneity estimates (I^2^ and Cochrane’s Q test) in the forest plots. A range of sensitivity analyses were also performed. Since Begg’s test for publication bias can be underpowered, we also reported bias-corrected estimates from Tweedie and Duval’s trim and fill method. Leave-one-out sensitivity analyses were performed to consider how dependent conclusions were to any individual study. Meta-regression models, which explored possible sources of heterogeneity, are reported in the manuscript whenever heterogeneity was significant (p<0.05) or substantial (I^2^ >0.25), otherwise in online supplementary material. Characteristics considered were: mean participant age, mean outcome biomarker levels at baseline, degree of intervention effectiveness for sedentary behaviour (intervention effect on overall sedentary time in h/day), intervention duration (≤3 months/3–6 months />6 months) and study quality (RoB scores). Unadjusted and age-adjusted models were reported.

## Results

### Systematic review

#### Study inclusion

**Figure 1:**
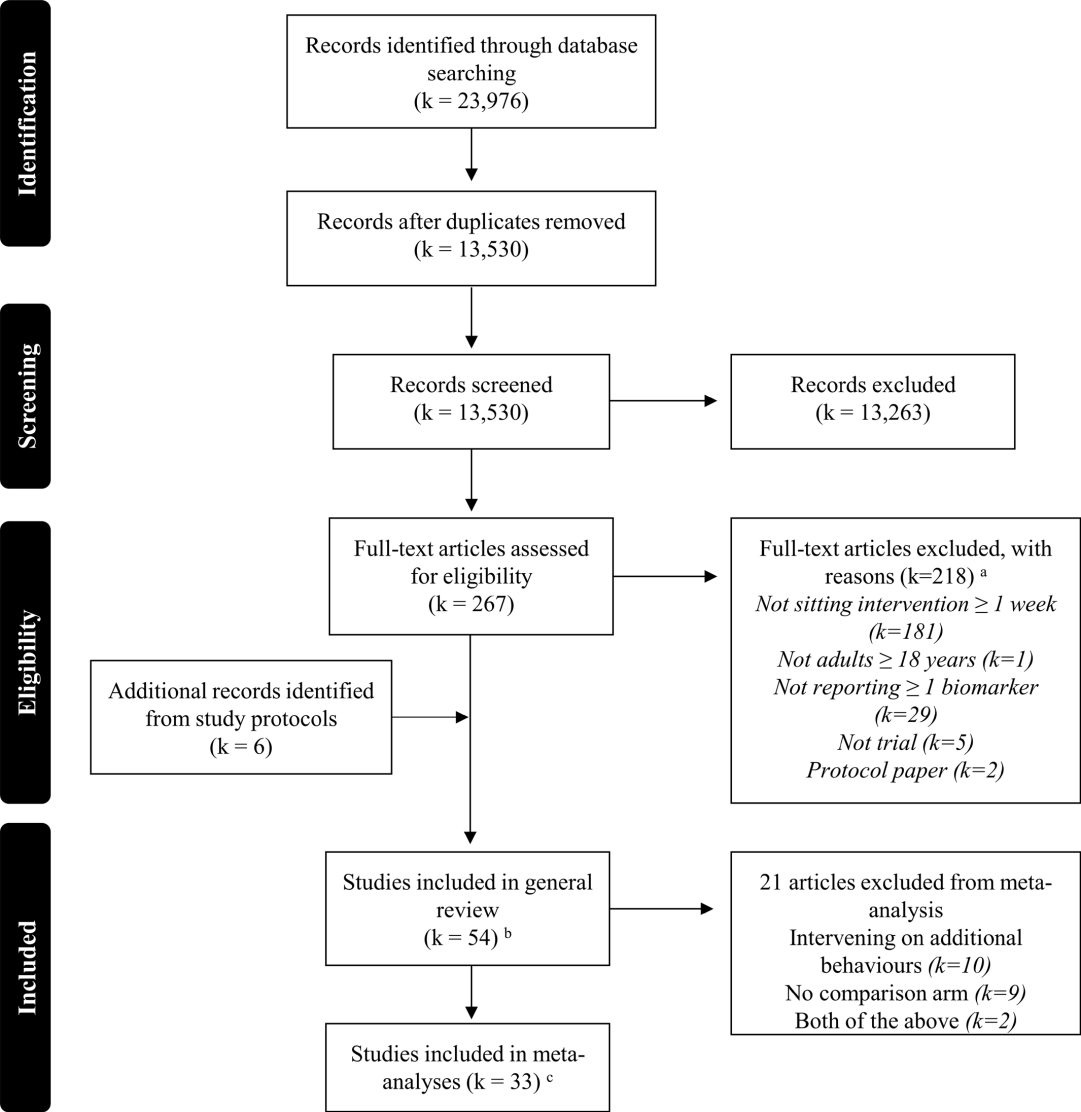
PRISMA diagram of the literature search results. ^a^ Exclusion criteria were applied in the following order: (1) not sitting intervention ≥1 week, (2) not a human study, (3) not English language, (4) not full-length publication, (5) not reporting ≥1 biomarker, (6) not randomised, quasi-randomised or pre–post trial, (7) not adults ≥18 years. ^b^ Two articles were identified for one study (Balducci *et al*,^47^ Balducci *et al* 72). ^c^ k=32 for anthropometry measures; k=25 for blood pressure measures; k=22 for glucose measures; k=24 lipid measures; k=4 inflammatory measures. PRISMA, Preferred Reporting Items for Systematic Reviews and Meta-Analyses.

Figure 1 shows the PRISMA flow diagram. In total, 23 976 articles were identified. Most were rejected at abstract screening, with 267 articles screened as full text. The criteria mostly excluded studies on the basis they were not ≥7 days sedentary behaviour interventions (181/218). Fifty-four studies (55 articles) were included in the systematic review.^33–87^

The population, design and intervention characteristics of the studies are reported in online supplementary table S3 and aggregated in table 1. Collectively, the 54 studies involved 6330 participants (48% women) with sample sizes usually <100 (k=36) and occasionally >200 (k=8), ranging between 12 and 1113. Study populations were recruited from developed nations, mostly English-speaking (table 1) usually from North America (k=20, predominantly the USA), Europe (k=19), Australia (k=10) and occasionally from Asia (k=4) or Africa (k=1). From what little and inconsistent data was reported on ethnicity, plus the study locations, we infer that most study participants were likely Caucasian or ‘white’ (variously defined) with a smaller number identifying as African American or African, Hispanic and Asian ethnicities. Study mean ages ranged from 23 years^74^ to 71 years.^51^ Typically, the studies recruited participants from the general population (k=26) or a population with a chronic disease risk factor (k=17) (generally overweight and/or obesity, occasionally in conjunction with another risk factor). Clinical conditions^36 37 39 44 48 59 60 71 72 81 84^ were seldom targeted for recruitment; of these only some conditions were pertinent to cardiovascular health. Many studies (k=25) used screening to recruit participants at risk for high sedentary behaviour based on their job (eg, office or desk-based work) and/or their reported behaviours, while 20 studies screened based on physical activity, and 11 studies screened on both behaviours.

10.1136/bjsports-2019-101154.supp3 Supplementary data

**Table 1:** Summary of the study population, design and intervention characteristics of adult sedentary behaviour interventions ≥7 days with biomarker outcomes

Characteristic		Count*	Detail
Population / study characteristics (54 studies)			
Sample size	Median n / study	57	Lowest=12, highest=1113
	Total n	5217	Female n=3065 (48.4%), male n=3265 (51.6%)
Location (continent and country: ISO 2-digit country codes)	North America	20	USA 16, CA 4
	Europe	19	UK 5, DK 4, SE 3, ES 2, NL 1, DK & GL 1, FI 1, IT 1, UK & NL & NO & PT 1
	Australia	10	
	Asia	4	TW 2, CN 1, JP 1
	Africa	1	ZA 1
Ethnicity	>50% Caucasian / ‘white’	18	
	<50% Caucasian / ‘white’	2	
	Not reported	33	
Clinical population	Clinical condition	11	T2D 4, cancer 2, rheumatoid arthritis 2, obstructive sleep apnoea 1, intellectual disability 1, coronary artery disease 1
	Clinical risk factors only	17	Adiposity 14, chronic disease risk (+adiposity) 3
	Healthy / general	26	
Screening	Sedentary job / behaviour	25	11 also screened for PA, 14 did not
	Physical activity (PA)	30	11 also screened for PA, 19 did not
Study design	Randomised controlled trial	39	8 cluster / 31 individually randomised 34 parallel group / 3 crossover / 1 other †
	Non-randomised controlled trial	5	1 cluster / 4 individually allocated 5 parallel group / 0 crossover
	Multiarm (no controls)	5	1 cluster randomised / 4 individually randomised
	Pre–post (single arm)	5	
Primary outcomes	Includes sedentary	22	
	Includes biomarker(s)	10	
	Includes both	1	
	Includes neither	21	PA 8 / PA and diet 1, unstated 6, feasibility 5, fitness 1
Intervention characteristics (56 interventions)			
Duration	3 months or less	28	
	>3 to 6 months	16	
	>6 months	12	
Setting	Workplace	27	
	Community	18	
	Other	11	Hospital 5, primary care 4, domestic 1, education 1
N components	Multicomponent	34	Workplace 13, community / other 21
	Single component	22	Workplace 14, community / other 8
Components‡	Counselling / education	41	Workplace 13, community / other 28
	Environmental modification	34	Workplace 26, community / other 8
	Prompting	12	Workplace 6, community / other 6
	Structured 'activity'	5	Workplace 2, community / other 4
	Device self-monitoring	23	Workplace 5, community / other 18
	Device social comparison	7	Workplace 3, community / other 4
	Financial incentives	2	Workplace 1, community / other 1
Sedentary targets / messaging‡	Domain specific message	29	
	Accumulation	21	
	Quantitative volume target	14	

*Count out of 54 studies or 56 interventions as indicated in the table unless other statistic is mentioned (eg, median).

†Almost a randomised controlled trial (parallel groups) except re-enrolled some controls into the intervention on completion.

‡Not mutually exclusive (interventions can have multiple components, multiple messages).

T2D, type 2 diabetes.

#### Interventions

The 54 studies delivered 56 sedentary behaviour interventions, mostly in the workplace (k=27) or community (k=18) settings, with healthcare (k=9), domestic (k=1) and educational settings (k=1) being less common (table 1, details in online supplementary table S3). Workplace interventions were about half multicomponent (k=13) and half single component (k=14), while interventions in the remaining non-workplace settings were mostly multicomponent (k=21, 72%). Workplace interventions almost all used environmental modification (k=26), commonly used counselling/education (k=13), sometimes used device self-monitoring (k=5), device-based social comparison (k=3), prompting via devices or SMS (k=6) and structured activity sessions (k=2). By contrast, non-workplace interventions almost always used some form of counselling/education (k=29), commonly used device self-monitoring (k=18) and occasionally used environmental modification (k=8), prompting (k=6), structured activity (k=4) and financial incentives (k=1). The extent of education or counselling was also highly variable, ranging from brief advice to theoretically grounded behavioural counselling.

The interventions varied in how they considered sedentary behaviour. Diverse behaviours were promoted as replacements for sedentary behaviour: primarily standing, walking or other stepping, but also sometimes pedalling, ‘incidental’ exercise (likely predominantly ‘light’ activities), activities of moderate or greater intensity and sometimes resistance exercise (online supplementary table S3). Sedentary behaviour targets seldom were domain specific, referenced accumulation patterns or set quantitative guidelines on sedentary time (table 1). Primary outcomes included sedentary behaviour in 23 studies, biomarkers in 11, and included neither in 20, instead being unstated (k=6), focused on feasibility (k=5) or involving physical activity with or without other outcomes (k=9).

#### Evaluation of biomarker indicators of cardiovascular health

The biomarkers selected for review are shown in table 2. Biomarker outcomes nearly always included indicators of body anthropometry (k=52 studies), and often included indicators of blood pressure (k=37), lipid metabolism (k=33) and glucose metabolism (k=31). Four studies reported on C reactive protein.^72 73 75 87^ Other inflammatory markers, such as tumour necrosis factor α or interleukin-6, were not found among the reported outcomes.

**Table 2:** Biomarkers reported as outcomes in 54 studies of adult sedentary behaviour interventions ≥7 days

Outcomes	Studies	Detail	Quality factors
**Body anthropometry**	52		
Body weight*	45	45 wt*, 39 body mass index BMI*	Objective† / self-report: 44/1
Waist circumference*	37	37 circumference*, 2 waist:hip ratio	Objective† / self-report: 36/1
Other body measurements	9	7 hip circumference, 1 neck circumference, 2 sagittal abdominal diameter	
Body composition	25		BIA: 12 DXA: 5 BIS: 2 BADP: 3 Skinfold(s): 2 Unreported: 1
Total fat	25	20 percentage of body weight*, 11 mass*	
Total fat-free or lean	13	12 percentage of body weight, 1 mass*,	
Other	5	fat mass or % (4 truncal, 1 arm, 1 leg, 1 android %, 1 gynoid %); fat-free mass or % (1 arm, 1 leg); 1 skeletal muscle mass; 1 visceral fat area	
**Blood pressure (BP) regulation**	37		
Resting BP *	37	37 systolic*, 36 diastolic*, two mean arterial BP	Objective† / self-report: 36/1
Ambulatory BP	0	–	–
Heart rate	5	3 resting, 2 non-resting	Objective† / self-report: 5/0
Detailed vascular health measures	3	1 flow mediated dilation, 1 carotid intima media thickness, 1 aortic augmentation index, 1 subendocardial variability, 1 pulse wave velocity	Objective† / self-report: 3/0
**Glucose metabolism**	31		
Fasting glucose*	27		Venous / capillary: 20/7
Fasting insulin*	13		
HOMA / HOMA-2	7	6 HOMA-IR, 2 HOMA-%B, 1 HOMA2-%B, 1 HOMA2-%S	
Postprandial glucose / insulin	4	4 postprandial glucose, 1 postprandial insulin, 1 insulin AUC, 1 glucose AUC, 1 C-ISI	Venous / capillary: 4/0 Duration: all 2 hours test
C-peptide	0		–
HbA1c*	17	15 HbA1c, 2 'estimated average glucose' reported as HbA1c	Venous / capillary: 15/2
**Lipid metabolism**	33		
Cholesterol levels or ratios	33	29 total*, 28 HDL*, 24 LDL*, 1 VLDL, 1 non-LDL, 5 total/HDL, 2 LDL/HDL	Venous / capillary: 25/8 fasted / insufficient / non-fasted state: 25/1/7
Triglycerides*	32		
Other	3	1 cholesterol diameter; 1 lipoprotein lipase; 3 apolipoproteins (APO): 3 APO-A1, 3 APO-B, 2 APO-A1/APO-B	
**Inflammation**	4		
C reactive protein (CRP)	4	2 CRP; two high-sensitivity CRP	Venous / capillary: 4/0 fasted / insufficient / non-fasted state: 4/0/0
Other: TNF-α, IL-6	0		–

Data were extracted from the earlier paper related to this study Balducci 2017 when it was not reported in the 2019 paper (body fat percentage; fat-free mass; BMI; fasting insulin; HOMA).

*Outcome included in the meta-analyses: was reported in >5 of the 33 studies eligible for the effectiveness meta-analyses (had control arm, no additional relevant intervention provided apart from active behaviours).

†Measured objectively by research staff.

AUC, area under the curve; BADP, body air displacement plethysmography; BIA, multifrequency bioimpedance analysis; BIS, bioelectrical impedance spectroscopy; BMI, body mass index; C-ISI, composite insulin sensitivity index; DXA, dual X-ray absorptiometry; HDL, high-density lipoprotein; HOMA-2, revised homeostatic model assessment; HOMA, homeostatic model assessment; IL-6, interleukin 6; LDL, low-density lipoprotein; TNF-α, tumour necrosis factor α; VLDL, very-low-density lipoprotein.

Of the anthropometric indicators, the most commonly reported were weight (k=45) or BMI (k=39), followed by waist circumference (k=37). These were almost always collected objectively by staff. Body composition outcomes were collected mostly using multifrequency bioimpedance analysis (k=12) or reference-grade standards: dual X-ray absorptiometry (k=5) or body air displacement plethysmography (k=3). Occasionally, other methods were used (k=4). Studies typically reported on body fat (k=25) (most commonly as percentage of body weight), and occasionally fat-free, lean or muscle mass (k=13). Thus, fewer studies were able to assess changes to specific tissues (eg, fat, lean tissue) or anatomical sites (eg, truncal fat, measured in four studies).

Blood pressure was generally assessed with resting blood pressure (k=37), which was typically reported separately as systolic (k=37) and/or diastolic blood pressure (k=36) and as mean arterial pressure in two studies^57 82^ (table 2). Usually, staff measured blood pressure, with participants reporting values from home monitors in one study.^46^ Ambulatory blood pressure was not reported. Detailed biomarkers of vascular health (eg, endothelial dysfunction, arterial stiffness) were seldom collected. One study reported on flow mediated dilatation, carotid artery intima media thickness, aortic augmentation index and sub endocardial variability.^53^ Resting heart rate was collected in three studies.^62 69 73^

Of the glucose metabolism indicators (table 2), most (k=27) reported on fasting glucose, with only 13 reporting fasting insulin, and 7 reporting composite indicators of beta-cell function or insulin resistance (ie, measures from homeostatic model assessment, HOMA or HOMA-2). Seventeen studies reported on overall glucose control (HbA1c expressed in various forms), while four studies reported effects on postprandial glucose and/or insulin^54 58 71 83^ and none reported on c-peptide. While venous blood draws were the norm for collecting fasting values (k=20 studies), lower quality fingerstick capillary measures were occasionally used (k=7). None of the studies reported outcomes from continuous glucose monitoring.

The most commonly reported lipid markers were: triglycerides (k=32); total cholesterol (k=29); high-density lipoprotein (HDL) cholesterol (k=28) and, low-density lipoprotein (LDL) cholesterol (k=24) (table 2). These markers are reported widely in the context of cardiovascular risk. Studies occasionally reported VLDL cholesterol,^69^ non-LDL cholesterol^40^ or cholesterol ratios.^40 42 43 60 70^ Three studies reported on apolipoproteins (APOA1, APOB and their ratio^38 70 73^) and one reported on the diameter of various types of cholesterol.^38^ None of the studies mentioned performing detailed profiling of lipid classes or subclasses.

#### Study designs

Very few studies (k=5) used a single-group pre–post study design (table 1)^46 51 63 69 78^; most used two or more groups (k=49). Usually the additional group (or groups) facilitated testing effectiveness against a no-intervention or attention control comparison arm (k=44, with 39 randomised) or occasionally only allowed for comparison of alternate interventions (k=5, with five randomised).^34 44 50 67 84^ Most studies (k=42) intervened for 6 months or less (shortest=2 weeks) while few (k=10) intervened for 12 months or longer^43 44 46 59 60 63 72 73 76 87^ (longest=36 months). Only nine studies referred to evaluation of maintenance of effects following withdrawal of intervention or intervention contact.

### Meta-analysis

#### Study inclusion

Of the 44 controlled intervention studies, 33 studies (34 interventions) were eligible for the meta-analyses (11 studies had provided diet intervention). For the 15 biomarkers that met the inclusion criteria (table 2), the number of studies providing data and able to be included ranged from 6 for fat mass to 25 for body weight and blood pressure, and these studies collectively represented anywhere between 724 and 2076 participants.

#### Risk of bias

RoB overall is reported in online supplementary table S6. To simplify reporting, criteria scored for groups of outcomes with similar concerns underlying their bias risk (eg, missing data, measurement): anthropometric and blood pressure outcomes, glucose metabolism outcomes and lipid metabolism outcomes. Overall RoB was high (≥1 criteria was ‘high’ risk) in 10 studies (30%), unclear (ie, 0 ‘high’ risk and ≥1 ‘unclear’ risk) in 17 (52%) studies and ‘low’ (ie, all ‘low’ risk) in 6 studies (18%). The most common contributor to a ‘high’ RoB rating related to the randomisation process (k=6, 18%),^58 64 68 76 79 87^ (ie, use of non-random methods). Four studies were also rated as ‘high’ RoB due to deviations from intended interventions (data not analysed according to intention-to-treat principles^40 58 74 79^) and missing outcome data.^76 79 83 87^ An unclear risk level was typically assigned based on inadequate reporting of randomisation (k=12), concerns with missing outcome data (k=11) and/or bias in measurement of the outcome (k=9). Low risk was still permitted with lack of blinding, given the context (behavioural intervention) in which allocation is impossible to conceal from participants and is generally known to staff, and in which outcomes are collected objectively.

10.1136/bjsports-2019-101154.supp6 Supplementary data

#### Effectiveness of sedentary behaviour interventions for biomarker outcomes

Effects on biomarkers were evaluated in the context of interventions that had displayed overall sedentary time improvements net of control that were mostly moderate (k=12, 30 to <60 min/day), otherwise strong (k=9, ≥60 min/day) or small (15 to <30 min/day, k=8) or occasionally almost zero (k=3, –15 to <15 min/day). Effects ranged from +11.3 to −132 min/day (see online supplementary table S4). Table 3 shows the pooled effects on biomarkers for the main analyses and sensitivity analyses. Begg’s tests were all p≥0.05 (online supplementary table S7).

10.1136/bjsports-2019-101154.supp4 Supplementary data

10.1136/bjsports-2019-101154.supp13 Supplementary data

**Table 3:** Pooled intervention effects on biomarkers: controlled trials of 34 adult sedentary behaviour interventions ≥7 days

Outcome	Main findings					Publication-bias corrected	Leave-one-out sensitivity analysis	
	k	n	All studies				Most benefit	Least benefit
			I^2^, P value	Pooled effect (95% CI)	P value	Pooled (95% CI)	Pooled effect (95% CI)	Pooled effect (95% CI)
Weight, kg	25	1839	23.6%, p=0.142	−**0.56 (−0.94 to −0.17**)	**0.005**	n/a	−**0.63 (−1.03 to −0.23**)*	−**0.47 (−0.75 to −0.18**)†
Body mass index, kg/m^2^	24	1843	0.0%, p=0.804	−0.07 (−0.16 to 0.03)	0.167	−0.08 (−0.17 to 0.02)	−0.10 (−0.20 to 0.01)‡	−0.04 (−0.14 to 0.06)§
Waist circumference, cm	19	2076	**45.8%, p=0.016**	−**0.72 (−1.21 to −0.22**)	**0.004**	−**1.00 (−1.51 to −0.49**)	−**0.95 (−1.38 to −0.51**)¶**	−**0.61 (−1.20 to −0.01**)¶††
Body fat, %	16	1618	5.5%, p=0.390	−**0.26 (−0.50 to −0.02**)	**0.034**	−**0.37 (−0.65 to −0.10**)	−**0.37 (−0.61 to −0.12**)‡	−0.17 (−0.43 to 0.09)**
Fat mass, kg	6	724	26.6%, p=0.235	−0.33 (−0.74 to 0.08)	0.116	n/a	−**0.42 (−0.73 to −0.10**)‡‡	−0.23 (−0.63 to 0.16)§§
Fat-free mass, kg	7	1011	**72.7%, p=0.001**	0.00 (−0.52 to 0.53)	0.992	0.48 (−0.02 to 0.98)	0.12 (−0.40 to 0.65)‡	−0.25 (−0.57 to 0.06)¶**
Blood pressure, mm Hg								
Systolic	25	1932	8.6%, p=0.341	−**1.05 (−2.08 to −0.02**)	**0.045**	n/a	−**1.42 (−2.38 to −0.45**)‡	−0.75 (−1.81 to 0.31)¶¶
Diastolic	25	1932	**52.6%, p=0.001**	−0.69 (−1.69 to 0.32)	0.180	n/a	−0.92 (−1.86 to 0.02)***	−0.36 (−1.28 to 0.56)†††
Glucose, mM	19	1518	**45.5%, p=0.017**	−0.03 (−0.11 to 0.05)	0.526	−0.04 (−0.13 to 0.05)	−0.05 (−0.11 to 0.02)¶‡‡‡	−0.01 (−0.11 to 0.09)¶¶
Insulin, pM	10	1102	**64.0%, p=0.003**	−**1.42 (−2.82 to -0.02**)	**0.047**	−1.03 (−2.48 to 0.42)	−**4.13 (−7.48 to -0.78**)§§§	−0.45 (−1.60 to 0.69)¶*
HbA1c, %	9	892	**72.9%, p=0.000**	−0.10 (−0.22 to 0.03)	0.129	−0.03 (−0.16 to 0.09)	−0.14 (−0.29 to 0.01)¶¶¶	−0.05 (−0.17 to 0.07)§§
Cholesterol, mM								
Total	23	1798	**54.1%, p=0.001**	−0.06 (−0.16 to 0.04)	0.213	−**0.10 (−0.20 to −0.00**)	−0.08 (−0.18 to 0.02)‡‡‡	−0.03 (−0.11 to 0.05)¶****
High-density lipoprotein	22	1760	22.5%, p=0.168	**0.04 (0.02 to 0.07**)	**<0.001**	**0.05 (0.02 to 0.07**)	**0.05 (0.03 to 0.07)§**	**0.03 (0.01 to 0.06)¶¶**
Low-density lipoprotein	20	1660	0.0%, p=0.690	−0.02 (−0.07 to 0.04)	0.562	−0.01 (−0.07 to 0.05)	−0.03 (−0.09 to 0.03)‡‡‡	−0.00 (−0.06 to 0.06)††††
Triglycerides, mM	23	1742	**49.0%, p=0.005**	−0.02 (−0.09 to 0.04)	0.496	−0.06 (−0.13 to 0.01)	−0.04 (−0.10 to 0.03)‡‡	−0.01 (−0.07 to 0.06)§§

k, n = number of interventions, number of individuals (sum of n analysed in each included study).

Boldface indicates pooled effect is p<0.05.

*Omitted Healy *et al* (2017).

†Omitted Ashe *et al* (2015).

‡Omitted Maylor *et al* (2018).

§Omitted Pesola *et al* (2017).

¶Heterogeneity no longer p<0.05 in leave-one-out sensitivity analysis.

**Omitted Danquah *et al* (2017).

††Omitted Puig-Ribera *et al* (2015).

‡‡Omitted Healy *et al* (2013).

§§Omitted Kallings *et al* (2017).

¶¶Omitted Butler *et al* (2018).

***Omitted Mantzari *et al* (2018).

†††Omitted Lin *et al* (2017).

‡‡‡Omitted Taylor *et al* (2016) (Computer intervention).

§§§Omitted Balducci (2019).

¶¶¶Omitted Biddle (2015).

****Omitted Thomsen *et al* (2017).

††††Omitted Aadahl *et al* (2014).

n/a, not applicable.

#### Body weight and body composition

Consistent with the studies’ selection criteria, prior to intervention, participants had a weighted mean (±pooled SD) BMI of 25.4±3.2 kg/m^2^, with study means ranging from 22.1 kg/m^2^ in a workplace intervention with no weight screening criteria^68^ to 35.9 kg/m^2^ in a treadmill intervention for overweight/obese office workers.^56^ Baseline anthropometric values are summarised in table 4 (detail in online supplementary table S4). Pooled effects showed that the sedentary behaviour interventions tended to provide small improvements (net of control) in body anthropometry outcomes (table 3). Significant pooled effects in favour of intervention were seen regarding body weight (−0.56 kg, 95% CI -0.94 to 0.17), waist circumference (−0.72 cm, 95% CI -1.21 to 0.22), body fat percentage (−0.26%, 95% CI -0.50% to 0.02%), with a tendency towards reduced fat mass (−0.33 kg, 95% CI −0.74 to 0.08) and no large or significant effect on fat-free mass (0.00 kg, 95% CI −0.52 to 0.53). Effects on BMI were in a similar direction to those for body weight, but not statistically significant (−0.07 kg/m^2^, 95% CI −0.16 to 0.03). Forest plots for body weight and body composition are shown in online supplementary figure 1–6. Small-study effects did not lead to overstated findings, as the original findings were no more favourable than the trimmed and filled results. Also, no single study seemed to overly influence the conclusions, as improvements observed were always still present to some degree in the leave-one-out sensitivity analyses.

10.1136/bjsports-2019-101154.supp7 Supplementary data

10.1136/bjsports-2019-101154.supp8 Supplementary data

10.1136/bjsports-2019-101154.supp9 Supplementary data

10.1136/bjsports-2019-101154.supp10 Supplementary data

10.1136/bjsports-2019-101154.supp11 Supplementary data

10.1136/bjsports-2019-101154.supp12 Supplementary data

**Table 4:** Average biomarker characteristics prior to intervention in controlled trials of 34 adult sedentary behaviour interventions ≥7 days with biomarker outcomes

	k	n	Weighted mean±pooled SD	Study means (min–max)	Study with lowest mean	Study with highest mean
Weight, kg	29	2456	71.3±10.6	62.2–99.6	Alkhajah *et al* 68	MacEwen *et al* 40
Body mass index, kg/m^2^	34	3186	25.4±3.2	22.1–35.9	Alkhajah *et al* 68	Schuna *et al* 56
Waist circumference, cm	21	2630	83.9±7.7	74.4–111.4	Butler *et al* 74	MacEwen *et al* 40
Body fat, %	18	2050	28.1±5.4	24.5–45.5	Dunning *et al* 75	Kozey Keadle *et al* 58
Fat mass, kg	7	753	25.3±7.7	18.4–32.3	Alkhajah *et al* 68	Kallings *et al* 70
Fat-free mass, kg	8	1252	40.0±6.7	44.1–56.5	Alkhajah *et al* 68	Balducci *et al* 72
Systolic BP, mm Hg	25	2461	110.0±10.5	109–142	Dunning *et al* 75	Maxwell-Smith *et al*
Diastolic BP, mm Hg	25	2457	68.4±6.5	69–86	Peterman *et al* 83	MacEwen *et al* 40; Maxwell-Smith *et al* 40
Glucose, mM	19	1975	4.7±1.0	4.1–7.6	Peterman *et al* 83	Balducci *et al* 72
Insulin, pM	11	1495	51.5±44.1	37.1–133.0	Dunning *et al* 75	Kozey Keadle *et al* 58
HbA1c, %	11	1308	4.4±0.6	4.9–7.4	Kallings *et al* 70	Balducci *et al* 72
Total cholesterol, mM	24	2292	4.3±0.6	4.0–5.5	Peterman *et al* 83	Kallings *et al* 70
HDL cholesterol, mM	22	2232	1.2±0.4	1.1–1.8	Peterman *et al* 83	Pesola *et al* 38
LDL cholesterol, mM	21	2142	2.5±0.8	2.5–3.3	Peterman *et al* 83	Kallings *et al* 70
Triglycerides, mM	23	2202	1.1±0.5	0.9–1.9	Alkhajah *et al* 68	Kozey Keadle *et al* 58

BP, blood pressure; HDL, high-density lipoprotein; k, number of interventions; LDL, low-density lipoprotein; n, number of participants.

Body weight and body fat percentage showed little evidence of heterogeneity (I^2^ <25%; p≥0.05) with slightly more substantial (but non-significant) heterogeneity seen for fat mass and significant heterogeneity seen for waist circumference and fat-free mass. The heterogeneity in effects on fat-free mass was completely attenuated (I^2^=0.0, p=0.790) by omitting a single study.^45^ Omission of this same study partially attenuated heterogeneity in effects on waist circumference (I^2^=19.9%, p=0.217). Further exploration of the heterogeneity via meta-regression (table 5) did not show any significant predictors of effects on waist circumference. The largest effects and the smallest residual heterogeneity were seen for RoB scores (residual I^2^=22.6%, p=0.192), with effects stronger by just over 1 cm in studies with high versus low RoB. Meta-regression results for the outcomes not displaying substantial or significant heterogeneity are shown in online supplementary table S8.

10.1136/bjsports-2019-101154.supp15 Supplementary data

**Table 5:** Associations of study characteristics with intervention effects on cardiometabolic biomarkers (meta-regression)

	Unadjusted				Age adjusted			
	k, n	b (95% CI)	P value	I^2^, p value	k, n	b (95% CI)	P value	I^2^, p value
**Waist circumference, cm**	**19**, **2076**			**45.8%, p=0.016**	**19**, **2076**			**45.8%, p=0.016**
Mean baseline age, per 10 y	18, 2050	−0.50 (−1.18 to 0.17)	0.144	51.6%, p=0.007		–	–	–
Mean baseline BMI, kg/m^2^	19, 2076	−0.04 (−0.25 to 0.16)	0.672	48.6%, p=0.011	18, 2050	−0.01 (−0.24 to 0.22)	0.910	54.5%, p=0.005
Mean baseline level	19, 2076	0.01 (−0.08 to 0.09)	0.856	44.9%, p=0.021	18, 2050	0.07 (−0.03 to 0.18)	0.182	45.9%, p=0.023
Sedentary effectiveness, h/day*	19, 2076	−0.62 (−1.42 to 0.18)	0.127	38.7%, p=0.048	18, 2050	−1.05 (−1.96 to −0.14)	0.023	40.3%, p=0.049
Duration (vs ≤3 months)	19, 2076		*0.896*	51.8%, p=0.007	18, 2050		*0.307*	57.7%, p=0.003
3–6 months		0.15 (−1.08 to 1.37)	0.816			0.92 (−0.69 to 2.52)	0.262	
>6 months		−0.36 (−2.28 to 1.57)	0.717			−0.25 (−2.28 to 1.78)	0.809	
Risk of bias (vs high risk)	19, 2076		*0.098*	22.6%, p=0.192	18, 2050		*0.109*	25.4%, p=0.174
Some concerns		−0.70 (−2.50 to 1.09)	0.441			−0.52 (−2.39 to 1.34)	0.582	
Low risk		−1.47 (−3.10 to 0.16)	0.077			−1.36 (−3.03 to 0.31)	0.110	
**Fat-free mass, kg**	**7**, **1011**			**72.7%, p=0.001**	**7**, **1011**			**72.7%, p=0.001**
Mean baseline age, per 10 years	7, 1011	0.14 (−0.60 to 0.88)	0.714	74.5%, p=0.001		–	–	–
Mean baseline BMI, kg/m^2^	7, 1011	0.07 (−0.18 to 0.32)	0.575	76.8%, p<0.001	7, 1011	0.07 (−0.24 to 0.38)	0.660	79.6%, p=<0.001
Mean baseline level	7, 1011	0.04 (−0.10 to 0.18)	0.540	75.0%, p=0.001	7, 1011	0.06 (−0.16 to 0.27)	0.618	79.4%, p=<0.001
Sedentary effectiveness, hour/day*	7, 1011	0.23 (−0.98 to 1.44)	0.711	77.1%, p<0.001	7, 1011	0.21 (−1.02 to 1.44)	0.738	79.4%, p=<0.001
Duration (>6 vs ≤3 months)†	7, 1011	0.06 (1.23 to 1.36)	0.922	77.1%, p<0.001	7, 1011	−0.14 (−1.86 to 1.58)	0.875	77.6%, p=0.001
Risk of bias (vs high risk)	7, 1011		*0.490*	69.9%, p=0.010	7, 1011		*0.619*	74.5%, p=0.008
Some concerns		0.72 (−0.53 to 1.98)	0.258			1.23 (−0.73 to 3.20)	0.219	
Low risk		0.12 (−1.10 to 1.34)	0.847			0.37 (−1.07 to 1.81)	0.615	
**Glucose, mM**	**19**, **1518**			**45.5%, p=0.017**	**19**, **1518**			**45.5%, p=0.017**
Mean baseline age, per 10 years	19, 1518	−0.01 (−0.08 to 0.07)	0.891	47.9%, p=0.012		–	–	–
Mean baseline BMI, kg/m^2^	19, 1518	0.01 (−0.01 to 0.03)	0.391	46.0%, p=0.017	19, 1518	0.01 (−0.01 to 0.04)	0.330	48.8%, p=0.012
Mean baseline level	18, 1497	−0.01 (−0.23 to 0.20)	0.908	45.8%, p=0.021	18, 1497	0.03 (−0.23 to 0.29)	0.819	46.0%, p=0.023
Sedentary effectiveness, h/day*	18, 1497	−0.02 (−0.21 to 0.16)	0.804	45.6%, p=0.021	18, 1497	−0.05 (−0.25 to 0.15)	0.632	46.0%, p=0.023
Duration (vs ≤3 months)	19, 1518		*0.611*	51.5%, p=0.007	19, 1518		*0.732*	54.0%, p=0.005
3–6 months		−0.00 (−0.19 to 0.19)	0.980			0.04 (−0.24 to 0.32)	0.769	
>6 months		−0.12 (−0.37 to 0.13)	0.345			−0.14 (−0.43 to 0.15)	0.355	
Risk of bias (vs high risk)	19, 1518		*0.424*	48.3%, p=0.014	19, 1518		*0.543*	49.7%, p=0.013
Some concerns		0.18 (−0.13 to 0.48)	0.256			0.21 (−0.12 to 0.54)	0.212	
Low risk		0.12 (−0.09 to 0.33)	0.267			0.17 (−0.09 to 0.42)	0.202	
**Insulin, pM**	**10**, **1102**			**64.0%, p=0.003**	**10**, **1102**			**64.0%, p=0.003**
Mean baseline age, per 10 years	10, 1102	0.76 (−2.63 to 4.15)	0.660	68.0%, p=0.002		–	–	–
Mean baseline BMI, kg/m^2^	10, 1102	0.20 (−1.09 to 1.48)	0.764	67.8%, p=0.002	10, 1102	0.02 (−1.60 to 1.65)	0.980	68.8%, p=0.002
Mean baseline level	10, 1102	0.15 (0.03 to 0.27)	0.018	61.1%, p=0.008	10, 1102	0.21 (0.12 to 0.31)	<0.001	0.0%, p=0.641
Sedentary effectiveness, hour/day*	10, 1102	1.65 (−6.15 to 9.45)	0.678	66.5%, p=0.002	10, 1102	3.51 (−6.34 to 13.35)	0.485	45.4%, p=0.077
Duration (vs ≤3 months)	10, 1102		*0.014*	50.8%, p=0.047	10, 1102		*0.268*	57.8%, p=0.027
3–6 months		2.14 (−7.20 to 11.48)	0.654			1.87 (−8.64 to 12.38)	0.728	
>6 months		7.87 (−0.21 to 15.95)	0.056			6.84 (−2.33 to 16.01)	0.144	
Risk of bias (vs high risk)	10, 1102		*<0.001*	11.9%, p=0.338	10, 1102		*0.211*	22.8%, p=0.255
Some concerns		−0.24 (−1.42 to 0.94)	0.692			−3.10 (−11.81 to 5.62)	0.486	
Low risk		−4.64 (−6.95 to −2.32)	<0.001			−5.60 (−11.33 to 0.14)	0.056	
**HbA1c, %**	**9**, **892**			**72.9%, p=0.000**	**9**, **892**			**72.9%, p=0.000**
Mean baseline age, per 10 years	9, 892	−0.10 (−0.18 to −0.02)	0.011	49.5%, p=0.054		–	–	–
Mean baseline BMI, kg/m^2^	9, 892	−0.01 (−0.05 to 0.03)	0.599	76.3%, p<0.001	9, 892	−0.03 (0.05 to –0.01)	0.004	0.0%, p=0.454
Mean baseline level	9, 892	−0.15 (−0.34 to 0.04)	0.127	76.3%, p<0.001	9, 892	−0.16 (−0.32 to 0.00)	0.055	49.1%, p=0.067
Sedentary effectiveness, hour/day*	9, 892	0.08 (−0.10 to 0.26)	0.379	69.5%, p=0.002	9, 892	−0.02 (−0.20 to 0.17)	0.862	56.4%, p=0.032
Duration (vs ≤3 months)	9, 892		*0.994*	78.0%, p<0.001	9, 892		*0.002*	24.7%, p=0.249
3–6 months		−0.02 (−0.35 to 0.32)	0.919			0.04 (−0.14 to 0.23)	0.641	
>6 months		−0.02 (−0.38 to 0.35)	0.932			−0.24 (−0.50 to 0.02)	0.069	
Risk of bias (vs high risk)	9, 892		*0.090*	75.4%, p<0.001	9, 892		*0.037*	59.4%, p=0.031
Some concerns		0.30 (−0.25 to 0.85)	0.287			0.28 (−0.23 to 0.79)	0.286	
Low risk		0.50 (−0.02 to 1.02)	0.058			0.40 (−0.09 to 0.89)	0.113	
**Diastolic blood pressure, mm Hg**	**25**, **1932**			**52.6%, p=0.001**	**25**, **1932**			**52.6%, p=0.001**
Mean baseline age, per 10 years	25, 1932	−0.38 (–1.30 to 0.54)	0.421	54.4%, p<0.001		–	–	–
Mean baseline BMI, kg/m^2^	24, 1903	−0.01 (−0.32 to 0.30)	0.946	53.2%, p=0.001	24, 1903	0.03 (–0.31 to 0.36)	0.885	55.2%, p =<0.001
Mean baseline level	24, 1911	−0.16 (−0.44 to 0.12)	0.250	52.0%, p=0.002	24, 1911	−0.11 (−0.42 to 0.20)	0.492	52.5%, p=0.002
Sedentary effectiveness, hour/day*	23, 1882	−1.07 (−2.83 to 0.69)	0.232	52.9%, p=0.002	23, 1882	−1.69 (−3.40 to 0.02)	0.053	43.2%, p=0.019
Duration (vs ≤3 months)	25, 1932		*0.655*	56.2%, p<0.001	25, 1932		*0.712*	57.9%, p =<0.001
3–6 months		0.22 (−2.50 to 2.94)	0.874			0.40 (−2.64 to 3.43)	0.798	
>6 months		−1.20 (−3.97 to 1.57)	0.397			−1.14 (−4.19 to 1.92)	0.465	
Risk of bias (vs high risk)	25, 1932		*0.699*	55.1%, p<0.001	25, 1932		*0.629*	57.1%, p =<0.001
Some concerns		1.20 −2.05 to 4.45)	0.468			1.82 (1.90 to 5.53)	0.338	
Low risk		1.03 (−1.58 to 3.64)	0.439			1.35 (1.56 to 4.25)	0.363	
**Total cholesterol, mM**	**23**, **1798**			**54.1%, p=0.001**	**23**, **1798**			**54.1%, p=0.001**
Mean baseline age, per 10 years	23, 1798	−0.14 (−0.22 to -0.07)	<0.001	17.1%, p=0.233		–	–	–
Mean baseline BMI, kg/m^2^	23, 1798	0.01 (−0.02 to 0.04)	0.664	54.6%, p=0.001	23, 1798	0.01 (−0.01 to 0.03)	0.398	17.4%, p=0.233
Mean baseline level	23, 1798	−0.24 (−0.50 to 0.02)	0.066	45.7%, p=0.011	23, 1798	−0.05 (−0.30 to 0.21)	0.713	20.6%, p=0.195
Sedentary effectiveness, hour/day*	23, 1798	0.08 (−0.04 to 0.20)	0.206	40.3%, p=0.027	23, 1798	0.01 (−0.10 to 0.12)	0.812	19.5%, p=0.208
Duration (vs ≤3 months)	23, 1798		*0.573*	54.6%, p=0.001	23, 1798		*0.003*	20.4%, p=0.202
3–6 months		0.07 (−0.17 to 0.31)	0.577			0.06 (−0.12 to 0.24)	0.493	
>6 months		0.13 (−0.12 to 0.39)	0.306			0.11 (−0.08 to 0.30)	0.259	
Risk of bias (vs high risk)	23, 1798		*0.044*	47.2%, p=0.009	23, 1798		*<0.001*	13.7%, p=0.284
Some concerns		−0.34 (−0.65 to –0.04)	0.028			−0.21 (−0.48 to 0.06)	0.134	
Low risk		−0.11 (−0.39 to 0.16)	0.419			−0.05 (−0.29 to 0.19)	0.689	
**Triglycerides, mM**	**23**, **1742**			**49.0%, p=0.005**	**23**, **1742**			**49.0%, p=0.005**
Mean baseline age, per 10 y	23, 1742	−0.05 (−0.11 to 0.02)	0.149	51.3%, p=0.003		–	–	–
Mean baseline BMI, kg/m^2^	23, 1742	0.00 (−0.02 to 0.02)	0.962	50.3%, p=0.004	23, 1742	0.01 (−0.02 to 0.03)	0.667	52.4%, p=0.003
Mean baseline level	22, 1721	−0.20 (−0.63 to 0.22)	0.350	45.1%, p=0.014	22, 1721	−0.13 (−0.53 to 0.26)	0.508	31.7%, p=0.087
Sedentary effectiveness, h/day*	22, 1721	−0.06 (−0.17 to 0.05)	0.279	45.9%, p=0.012	22, 1721	−0.13 (−0.21 to –0.05)	0.001	0.0%, p=0.507
Duration (vs ≤3 months)	23, 1742		*0.645*	53.4%, p=0.002	23, 1742		*0.447*	55.7%, p=0.001
3–6 months		−0.07 (−0.24 to 0.10)	0.402			−0.05 (−0.24 to 0.14)	0.639	
>6 months		−0.06 (−0.25 to 0.13)	0.516			−0.07 (−0.28 to 0.13)	0.481	
Risk of bias (vs high risk)	23, 1742		*0.396*	53.6%, p=0.002	23, 1742		*0.485*	55.7%, p=0.001
Some concerns		−0.13 (−0.33 to 0.07)	0.218			−0.07 (−0.34 to 0.19)	0.587	
Low risk		−0.10 (−0.28 to 0.07)	0.245			−0.06 (−0.28 to 0.16)	0.573	

Table presents unstandardised regression coefficient (b) and 95% CI and p value from meta-regression of controlled trials of adult sedentary behaviour interventions ≥7 days. Italics indicates overall p value (omnibus test).

k=total number of interventions included and n=total number of individuals analysed in the included interventions, in the meta-regressions or main meta-analysis (boldface).

Residual heterogeneity (I^2^ and p from Cochrane’s Q test) with overall heterogeneity in the main meta-analysis shown in boldface.

*Estimated effectiveness of intervention on overall sedentary time (net of control).

†No studies in the 3–6 months duration category.

BMI, body mass index.

#### Blood pressure

Prior to intervention, participants had a weighted mean (±pooled SD) blood pressure of 110.0±10.5 mm Hg systolic and 78.4±7.1 mm Hg diastolic, indicating typically healthy levels, though with some studies attracting samples with average systolic blood pressure as high as 140 mm Hg or higher^55 70^ (table 3 and online supplementary table S4). Pooled effects showed a small significant reduction in systolic blood pressure (−1.05 mm Hg, 95% CI -2.08 to 0.02) and a smaller non-significant reduction in diastolic blood pressure (−0.69 mm Hg, 95% CI −1.69 to 0.32; table 3). Forest plots are shown in online supplementary figure S7 and online supplementary figure 8. Corrections for small-study effects had no effect on the results and pooled effects consistently reflected tendencies towards reduced blood pressure in the leave-one-out sensitivity analyses. Heterogeneity was minimal for systolic blood pressure (I^2^=8.6, p=0.341) but extensive for diastolic blood pressure (I^2^=52.6, p=0.001), and not explained by any single study. None of the variables in the meta-regressions (table 5) had significant associations with diastolic blood pressure or reduced the heterogeneity appreciably (residual I^2^ >50).

10.1136/bjsports-2019-101154.supp14 Supplementary data

#### Glucose metabolism

Prior to intervention, fasting glucose averaged 4.7±1.0 mM, indicating levels consistent with healthy metabolism or prediabetes rather than diabetes. However, the studies covered a diverse spectrum from 4.1 mM in a study of healthy adults^83^ to 7.6 mM in a study of type 2 diabetes patients aged 40–80 years.^72^ Baseline insulin and HbA1c levels averaged 51.5±44.1 pM and 4.4%±0.6% were also quite variable across studies (table 3 and online supplementary table S5). Pooled effects pointed to small benefits to glucose metabolism, which were statistically significant only for fasting insulin (−1.42 pM, 95% CI -2.82 to 0.02) and small non-significant tendencies towards lower fasting glucose (−0.03 mM, 95% CI −0.11 to 0.05) and HbA1c (−0.10%, 95% CI −0.22% to 0.03%). Forest plots are shown in online supplementary figure 9–11. Small-study effects may have overstated effects on insulin and HbA1c.

10.1136/bjsports-2019-101154.supp5 Supplementary data

10.1136/bjsports-2019-101154.supp16 Supplementary data

10.1136/bjsports-2019-101154.supp17 Supplementary data

10.1136/bjsports-2019-101154.supp18 Supplementary data

Glucose, insulin and HbA1c all showed substantial heterogeneity (I^2^=45.5 for glucose to I^2^=72.9 for insulin; p<0.05), which remained present in all the leave-one-out sensitivity analyses, except for glucose, where removing a single workplace study^49^ that had failed to elicit changes in sedentary behaviour markedly attenuated the heterogeneity (I^2^=28.4, p=0.126). Insulin outcomes were significantly beneficially associated with lower baseline levels, shorter intervention duration and higher RoB, with limited residual heterogeneity after accounting for RoB (I^2^=11.9, p=0.338); however, only the association with baseline level remained significant accounting for age (residual I^2^=0.0, p=0.641). Higher participant age significantly predicted enhanced HbA1c outcomes, and led to lower heterogeneity (residual I^2^=49.5, p=0.054) while in age-adjusted models, effects were significantly beneficially associated with higher BMI, longer intervention duration and lower RoB, and a borderline association with higher baseline levels. The model with age and BMI had no residual heterogeneity (I^2^=0.0, p=0.454).

#### Lipid metabolism

Prior to intervention, baseline levels averaged 4.3±0.6 mM total cholesterol, 1.2±0.4 mM HDL, 2.5±0.8 mM LDL and 1.1±0.5 mM triglycerides, with comparatively limited variation across studies relative to other biomarkers (table 4, online supplementary table S5). Small significant improvements in response to sedentary behaviour interventions were seen in HDL cholesterol (0.04 mM, 95% CI 0.02 to 0.07) alongside a small, non-significant improvement in total cholesterol (−0.06 mM, 95% CI −0.16 to 0.04) and very small, non-significant effects on LDL cholesterol (−0.02 mM, 95% CI −0.07 to 0.04), and triglycerides (−0.02 mM, 95% CI −0.09 to 0.04). Forest plots for cholesterol and triglycerides are shown in online supplementary figure S12 and online supplementary figure 13–15. Small-study effects if anything limited the effects seen for lipid metabolism, with trimmed-and-filled estimates all either larger or virtually unchanged, and with a significant effect on total cholesterol emerging (−0.10 mM, 95% CI -0.20 to 0.00).

10.1136/bjsports-2019-101154.supp19 Supplementary data

10.1136/bjsports-2019-101154.supp20 Supplementary data

10.1136/bjsports-2019-101154.supp21 Supplementary data

There was limited heterogeneity in outcomes concerning HDL and LDL cholesterol (I^2^ <25, p≥0.05) and more substantial and significant heterogeneity in total cholesterol (I^2^=54.1, p=0.001) and triglycerides (I^2^=49.0, p=0.005). Removing one study^36^ markedly lowered the total cholesterol heterogeneity (I^2^=21.1, p=0.183) while the same was not the case for triglycerides. Meta-regressions (table 5) showed significantly greater reductions in total cholesterol were seen with higher age, and higher RoB, with limited residual heterogeneity left after accounting for age (I^2^=17.1, p=0.233) while in age-adjusted models, significant predictors of greater reductions were shorter study duration and higher RoB. It appears multiple factors may have contributed to the heterogeneity in triglyceride outcomes. None of the variables significantly predicted effects on triglycerides and residual heterogeneity remained high in all models (residual I^2^=45.1–53.6). In age-adjusted models, less effectiveness in improving sedentary behaviour outcomes significantly predicted greater reductions in triglycerides (−0.13 mM, 95% CI -0.21 to 0.05), with very limited heterogeneity left when considering both these factors simultaneously (I^2^=0.0, p=0.507), which also involved excluding one study^74^ due to missing data.

## Discussion

Several reviews have reported on sedentary behaviour interventions in relation to sedentary behaviour outcomes^23 26 27^ and found them to be effective, to varying degrees. These reviews indicated success seemed to vary depending on factors including the focus on sedentary behaviour (alone vs in combination with other lifestyle behaviours) and the type of intervention (with multicomponent workplace interventions being particularly successful). The current systematic review with meta-analyses considered these interventions in the context of their effect on biomarkers of cardiometabolic health, finding a small body of evidence. In total, 54 studies were identified, with 33 eligible for the meta-analyses, and with 6–25 controlled interventions ultimately included in meta-analyses concerning body anthropometry, blood pressure and haemodynamics, glucose metabolism and lipid metabolism.

Broadly, the meta-analyses provided some support for small improvements in selected indicators of body anthropometry, blood pressure, glucose metabolism and lipid metabolism with intervention, with none of the outcomes tending to worsen with intervention. Specifically, significant improvements were seen in body weight, waist circumference, percentage body fat, systolic blood pressure, insulin and HDL cholesterol. For some outcomes, findings varied widely from study to study, while for others they were quite consistent, with heterogeneity ranging widely (I^2^=0.0–72.9). It may be the case that some types of interventions are effective (and others ineffective), and/or the interventions may be effective in some populations but not others. The sensitivity analyses and meta-regressions provided some insight into potential factors underlying some of the heterogeneous results. Sometimes a single study deviating from the general pattern appeared to be the issue, while other key factors (different for each outcome) tended to be due to participant age and BMI, study duration and RoB. There were very few studies with each characteristic; consequently, the CIs around effects were quite wide, and findings should not be taken to indicate non-significant predictors in the meta-regressions were unimportant. The low number of studies was also the reason stratified analyses were not performed to inform the effectiveness of specific types of interventions, for specific populations (eg, men, women, older adults and those with clinical conditions such as type 2 diabetes). Some potential success factors not able to be explored were ethnicity (poorly reported), sex, behaviour settings and dose response. Prior findings have sometimes suggested the biological responses to sedentary time may vary depending on the setting or context in which it occurs,^88 89^ by ethnicity,^90–92^ by sex^18 91 93 94^ and by the activity replacing sedentary time.^95–98^

The systematic review showed some key considerations for interpreting the effectiveness findings. The sedentary behaviour interventions performed were highly varied in terms of their setting, use of behavioural change components, and the degree of emphasis on sedentary behaviour; thus, the heterogeneous outcomes were not highly surprising. Also, some caution should be exerted in extrapolating findings to groups with limited or no representation in the evidence base. Evidence has mostly been collected from studies of Caucasian or ‘white’ populations (variously defined) of working age, often with overweight/obese BMI or waist circumference, with very limited representation of those with clinical conditions pertinent to cardiovascular health, such as type 2 diabetes. The short duration of most interventions may have influenced the degree of effectiveness observed in the meta-analyses; there was a paucity of studies intervening ≥12 months and including maintenance evaluations from which to consider sustainability or determine what may happen in the longer term. Previously, it has been reported that biomarker results have been more promising at 12 months compared with 3 months, despite sitting reduction being greatest at 3 months.^43^

To overcome the limitations of the current evidence base the next logical step would be individual patient data meta-analysis, with interventions collecting ‘dose’ data regarding sedentary behaviour and the activities that may replace it in the most harmonisable way possible, even if this is only possible in a subsample of participants. Ideally, the measurement should allow both calculation of some total dose (eg, in MET hours), as well as partial out time spent sedentary and in various alternative behaviours, delineated by intensity, posture and accumulation method (eg, sedentary/sitting, standing, light movement, moderate movement, vigorous movement and bouted vs non-bouted forms of the relevant behaviours). Such an approach may help to determine the populations for which each intervention may be effective, as well as ascertain which specific behaviours (if any) may achieve the greatest biomarker improvements.

Other key features identified within the current evidence base are the type, reporting (or lack thereof) and specificity/sensitivity of biomarker outcomes collected. For example, most of the biomarkers collected (eg, blood glucose, insulin, triglycerides and blood pressure) are subject to homeostatic regulation but were only measured in fasted or resting states. It is important to also evaluate how some sensitive biomarkers (without these limitations) that have fairly consistently responded beneficially in acute laboratory interventions lasting <7 days^9 11 12 99^ respond over longer intervention timeframes. Specifically, postprandial glucose, insulin, triglycerides and ambulatory blood pressure should be measured. Other understudied outcomes that are potentially useful to measure are: detailed markers of vascular haemodynamics and structure (eg, cardiovascular and cerebrovascular blood flow, flow-mediated dilatation and arterial stiffness)^99 100^; C-peptide; continuous glucose monitoring; postprandial lipids; lipid subclasses^101 102^; site-specific tissue samples (eg, muscle, adipose tissue) and additional intermediate biomarkers (such as those related to systemic metabolic/oxidative stress and inflammation).^9 100^ These outcomes could be collected in all participants or in subsamples, as they represent opportunities to detect changes that might otherwise be missed, and improve our understanding of shared risk factors and potential mechanistic pathways.

There were some caveats regarding the overall quality of the evidence. Trimmed-and-filled results mostly suggested publication bias did not affect findings, but the insulin finding may be overstated and some of the lipid findings understated. Inferences were sometimes made from a very small number of studies (especially regarding biomarkers of glucose and lipid metabolism), which is especially concerning with the findings varying so much between studies. The paucity of ‘low’ RoB studies is a limitation, though importantly most studies had an ‘unclear’ rather than a ‘high’ RoB and the meta-regressions did not usually show high RoB equated to the most promising results (if anything, findings showed the opposite).

## Conclusions

This systematic review with meta-analyses synthesised the body of work concerning the effectiveness of sedentary behaviour interventions on biomarkers of cardiometabolic risk, specifically: body anthropometry; blood pressure and related haemodynamics; glucose metabolism; lipid metabolism and inflammation. Consistent with evidence from prior observational research and acute laboratory-based experiments (<7 days) linking sedentary behaviour with cardiometabolic health,^8 11 12^ the evidence from ≥7 days interventions in free-living conditions showed small improvements in some cardiometabolic biomarkers. These biomarker improvements definitively occurred in response to interventions targeting sedentary behaviour (alone or alongside physical activity), but how they occurred in response to sedentary reductions and increases in various forms of physical activity remains unclear. Our review indicated that studies in clinical populations, ethnicities other than Caucasian or ‘white’ in predominantly Western countries, and evaluation of biomarkers of inflammation and postprandial metabolism are key areas for future research.

**What is already known**

- Recent reviews have demonstrated sedentary-reduction interventions are effective at modifying behaviour (reducing sitting).
- Observational and experimental research (mostly acute laboratory-based work) links both high volumes and prolonged periods of sedentary behaviour (sitting) with adverse health outcomes.
- However, less is known about the nature and extent of health effects with sedentary behaviour interventions over longer periods and under free-living conditions.

**What are the new findings**

- This review evaluated the evidence regarding the impact that interventions to reduce sedentary behaviour, alone or in combination with physical activity increases, may have on important indicators of cardiometabolic risk, when intervening for ≥7 days under free-living conditions.
- Available evidence for different outcomes ranged from 6 to 25 controlled trials. On average, these interventions led to modest improvements in selected indicators of body anthropometry, glucose and lipid metabolism, and blood pressure regulation, with no adverse effects observed.
- Potential improvements for future research were noted: more high-quality studies and interventions > 12 months; more population diversity (based on ethnicity, age, and clinical factors); more sensitive biological indicators; and, more studies evaluating vascular function and inflammation.
