# Supplementary material for: Effects of sedentary behaviour interventions on biomarkers of cardiometabolic risk in adults: systematic review with meta-analyses
Source: Br J Sports Med. 2020 Apr 8;55(3):144–54. doi: 10.1136/bjsports-2019-101154 (PMC7841485; doi:10.1136/bjsports-2019-101154)
Supplement: Supplementary data [file bjsports-2019-101154supp001.pdf]

Supplemental Material S1 Searches performed for Ovid MEDLINE(R) In-Process & Other Non-Indexed Citations, Ovid MEDLINE(R)

#	Searches	Results[a]
1	Sedentary Lifestyle/ or Television/ or Computers/ or Video Games/	0
2	(sitting or sedentary).tw.	5374
3	((reclining or lying) adj4 (time* or bout* or posture* or position*1)).tw.	264
4	((screen or screens or screenbased or TV or television* or computer* or device* or smartphone* or phone* or tablet* or ipad* or DVD or video*) adj4 (time* or hours or watch* or view*)).tw.	4377
5	((computer* or internet*) adj ("use" or "useage" or "usage" or behavio?r*)).tw.	543
6	((reading or gaming) adj3 time*).tw.	276
7	((videogam* or video game* or computer game* or electronic game*) adj5 (time* or bout* or hours or watch* or view*)).tw.	103
8	Automobile Driving/	0
9	((car or cars or automobile* or driving or commute* or commuting) adj3 time*).tw.	254
10	(physical* adj3 inactiv*).tw.	1158
11	((inactive or inactivity) adj3 (time* or bout* or hours)).tw.	102
12	(low adj2 energy expenditure*).tw.	34
13	(low adj2 physical activity*).tw.	357
14	or/1-13	11977
15	((cardiovascular or cardiometabolic or cardio-metabolic) adj2 risk*).tw.	10453
16	(metaboli* or dysmetaboli*).tw.	92550
17	body mass index/ or exp overweight/ or waist circumference/ or waist-hip ratio/	0
18	Body Weight/ or Weight Loss/ or Weight Gain/ or exp Adipose Tissue/ or Adiposity/ or body composition/ or body fat distribution/	0
19	exp Blood Pressure/ or exp Proinsulin/ or exp Adipokines/ or exp Somatomedins/ or exp Lipids/	0
20	Hemoglobin A, Glycosylated/ or Biological Markers/	0
21	C-Reactive Protein/ or Tumor Necrosis Factor-alpha/ or Interleukin-6/ or exp Fibrinogen/	0
22	(glycosuria* or hyperglyc?emi* or hyperinsulin* or hypertensi* or prehypertensi*).tw.	37777
23	(glucose or blood sugar* or glyco* or glyca* or glyce*).tw.	79568
24	(overweight or over-weight or obesity or obese or BMI or body mass index or body mass indicator*).tw.	49646
25	(waist circumference* or waist-hip ratio* or adiposity or adipose or fatty tissue*).tw.	13449
26	(weight adj1 (gain* or increase* or loss* or decrease* or reduc* or lose* or losing or fluctuat*)).tw.	16489
27	(body adj (fat or composition or weight)).tw.	20018
28	(blood pressure or diastolic or systolic).tw.	27762
29	(proinsulin* or insulin* or c-peptide* or adiponectin* or leptin* or resistin* or somatomedin*).tw.	28561
30	(lipid* or triglycerid* or triacylglycerol* or lipoprotein* or apolipoprotein* or apoprotein* or chylomicron* or diglycerid* or diacylglycerol*).tw.	49109
31	(dyslipid* or dyslipoprotein* or hyperlipid* or hyperlip?emi* or lip?emi* or lipid?emi* or hypercholesterol* or hyperlipoprotein* or hypertryglycerid*).tw.	8511
32	(HDL or LDL or VLDL or cholesterol*).tw.	19137

33	(biologic* marker* or biomarker* or immun* marker* or biochemical marker*).tw.	34917
34	(c-reactive or crp or fibrin* or hscrp or homocystein*).tw.	14516
35	(il6 or il-6 or interleukin-6 or tumo?r necrosis factor alpha or tnf-a or tnf-alpha).tw.	19760
36	((glycate* or glycosylate*) adj2 (hemoglobin* or haemoglobin*)).tw.	2598
37	(hb a1* or hba1*).tw.	4575
38	(HOMA or homeosta* model assessment*).tw.	2421
39	or/15-38	334401
40	movement/ or locomotion/ or walking/ or motor activity/ or exercise/ or resistance training/	0
41	physical exertion/ or physical fitness/	0
42	accelerometry/ or actigraphy/	0
43	(acceleromet* or accelero-met* or pedomet* or inclinomet* or actigraph* or acti-graph* or actimet* or acti-met*).tw.	3271
44	((activit* or movement* or motion or fitness) adj2 (monitor* or assess* or measure* or track* or sensor*)).tw.	10370
45	(physical* adj2 (fit* or activ* or train*)).tw.	14689
46	(ambulation* or ambulatory activit* or walk or walking).tw.	12556
47	((light or moderate or intensity) adj2 activit*).tw.	2040
48	(step or steps or stepping).tw.	76852
49	(sit-stand* or stand-step* or standing).tw.	10846
50	(GT1M or GT3X or GT3X+ or activPAL or Fitbit or GENEactiv or Jawbone or LUMOback or Fuelband or Omron).tw.	413
51	(Actiheart or Actical or Movemonitor or Tractivity or Yamax or Sensewear or Misfit or IDEEA or DirectLife or SmartShoe).tw.	700
52	(3dNX or ADXL322 or DynaPort MiniMod or EGAS device or GENEA or Minimod or RT3 device).tw.	9
53	(Digi-Walker or Caltrac or RT3 triaxial or Tracmor2 or Lifecorder or Walk4Life).tw.	13
54	(StepWatch or TriTrac or Axivity or BioTrainer or Activ Tracer or ActiWatch or ActivTracer or Mini-Motionlogger).tw.	54
55	or/40-54	122156
56	(randomized controlled trial or controlled clinical trial).pt.	307
57	(random* or quasirandom* or trial or placebo).tw. or clinical trial*.sh.	156307
58	cross-over studies/ or (cross-over or crossover).tw.	9949
59	or/56-58	162180
60	14 and 39 and 55 and 59	402
61	exp animals/ not humans.sh.	1
62	60 not 61	402
63	limit 62 to english language	399

[a]Results are displayed for the initial search (March 03 2017). Identical searches were performed, ultimately covering research up to February 16 2018.
