# Supplementary material for: Effects of sedentary behaviour interventions on biomarkers of cardiometabolic risk in adults: systematic review with meta-analyses
Source: Br J Sports Med. 2020 Apr 8;55(3):144–54. doi: 10.1136/bjsports-2019-101154 (PMC7841485; doi:10.1136/bjsports-2019-101154)
Supplement: Supplementary data [file bjsports-2019-101154supp002.pdf]

Supplemental Material S2 Rules used for extracting data for the meta-analyses

**Consideration**	**Rule** [a]
Quality control	One enters, another checks, discrepancies are discussed and resolved.
Intervention selection	The sedentary-behaviour only intervention in a factorial design. All sedentary behaviour interventions within a study for multiple differing interventions.<br>*NOTE: The only meta-analysed multi-arm studies were Taylor et al. 20xx (both included) and Kozey-Keadle et al. 2014 (only one intervention included).*
Endpoint selection	End of intervention. If unavailable, use interim results or results averaged across several follow-up endpoints (only if the authors indicate no difference between these follow-up endpoints.)<br>*NOTE: all estimates extracted were end of intervention except Balducci 2017 reported some outcomes not in Balducci 2019.*
Statistical conversions	$SE = (\text{Upper} - \text{Lower Limit})/(2 \times 1.96)$ and $SE = SD/\sqrt{n}$.
Unit conversions*	1 kg = 0.453592 pounds<br>1 inch = 2.54 cm<br>1 mM = 18 mg/dl (glucose)<br>1 mM = 38.67 mg/dl (cholesterol)<br>1 mM = 88.57 mg/dl (triglycerides)<br>1 µU/mL = 6 pmol/L insulin<br>NGSP HbA1c = (0.09148*IFCC HbA1c) + 2.152<br>eAG = 1.59 * NGSP HbA1c - 2.59<br>Percentage of baseline values: * baseline values / 100
Intervention effect extraction (minimum required data = intervention effect and its SE)	To maximise the amount of available data while retaining sufficient similarity for pooling. In order of preference extract:<br>*Intervention effects (between-group differences in change, in units)*<br>1. As reported<br>2. Calculated from each group's reported mean changes<br>3. Calculated from each group's reported means at baseline and follow-up (i.e., follow-up mean – baseline mean).<br>4. By requesting sufficient data to apply #1 or #2 from the authors<br>*Standard Error (SE) of intervention effect above*<br>1. From reported SE or 95% Confidence Intervals (CI)<br>2. By performing an independent samples t-test from within-group data, using standard deviations (SD) or SE of change<br>3. By requesting sufficient data to apply #1 or #2 from the authors<br>4. By performing an independent samples t-test of using (SD) within-group data, using standard deviations (SD) or SE at end of intervention.<br>*NOTE: Data were requested from 11 authors and 5 provided data while 6 did not respond or could not acquire the data, leading to the use of estimates from end-of-intervention SE for 4 studies and data being unreportable from 2 studies.*
Outcomes in review	This is a high-burden request. Contact authors and request only when a

but not in paper	very similar outcomes was reported (e.g., fat mass / percentage body fat, weight / BMI, cholesterol ratios / values) and request only from authors on the research team. *NOTE: This occurred for Alkahjah et al 2019, Healy 2013 and Healy 2017.*
Errors suspected	In order of preference<br>1. attempt to verify mathematically and resolve mathematically from other data reported<br>2. contact authors<br>*NOTE: Authors contacted were Ashe et al (2015) (large weight discrepancy; verified outcomes were correct); Biddle et al. (2015) (identical values for >1 outcome; provided new data); Mantzari et al (2019) (95% CI not logical; provided new data); Thomsen et al. 2016 and Thomsen et al. 2017 (impossible units mM HbA1c; responded was eAG mM, with the following conversion formula listed in unit conversions)*

[a] The same general procedures were applied in extracting intervention effects on sedentary behaviour outcomes, and baseline values of age and the biomarker outcomes, except that authors were not contacted to obtain further baseline data, only intervention effects.
