# Supplementary material for: Effects of sedentary behaviour interventions on biomarkers of cardiometabolic risk in adults: systematic review with meta-analyses
Source: Br J Sports Med. 2020 Apr 8;55(3):144–54. doi: 10.1136/bjsports-2019-101154 (PMC7841485; doi:10.1136/bjsports-2019-101154)
Supplement: Supplementary data [file bjsports-2019-101154supp006.pdf]

Supplemental Table S7 Assumption tests regarding publication bias

**Outcome**	**Begg's test**
Weight, kg	z = -0.21, p = 0.870
Body Mass Index, kg/m^2	z = -0.57, p = 0.602
Waist circumference, cm	z = 1.40, p = 0.162
Body fat, % of body weight	z = 0.68, p = 0.499
Fat mass, kg	z = 1.13, p = 0.260
Fat-free mass, kg	z = -1.20, p = 0.368
Systolic BP, mmHg	z = -0.49, p = 0.657
Diastolic BP, mmHg	z = -1.38, p = 0.183
Glucose, mM	z = 0.70, p = 0.484
Insulin, pM	z = 0.36, p = 0.721
HbA1c, %	z = -0.52, p = 0.754
Total cholesterol, mM	z = -0.71, p = 0.509
HDL cholesterol, mM	z = 0.08, p = 0.932
LDL cholesterol, mM	z = -0.68, p = 0.538
Triglycerides, mM	z = 1.35, p = 0.177
