# Supplementary material for: Effects of sedentary behaviour interventions on biomarkers of cardiometabolic risk in adults: systematic review with meta-analyses
Source: Br J Sports Med. 2020 Apr 8;55(3):144–54. doi: 10.1136/bjsports-2019-101154 (PMC7841485; doi:10.1136/bjsports-2019-101154)
Supplement: Supplementary data [file bjsports-2019-101154supp004.pdf]

Supplementary Table S6 Individual study risk of bias assessment for 1) anthropometric and blood pressure, 2) glucose, and; 3) lipid measures.

**No.**	**Study**	**Bias arising from the randomisation process**			**Bias due to deviations from intended intervention**			**Bias due to missing outcome data**			**Bias in measurement of the outcome**			**Bias in selection of the reported result**			**Overall bias**		
	Measure	***1***	***2***	***3***	***1***	***2***	***3***	***1***	***2***	***3***	***1***	***2***	***3***	***1***	***2***	***3***	***1***	***2***	***3***
1	Aadahl 2014	+	+	+	+	+	+	?	?	?	+	+	+	+	+	+	?	?	?
2	Alkhajah 2012	-	-	-	+	+	+	?	-	-	+	+	+	+	+	+	-	-	-
3	Ashe 2015	?			?			?			+			+			?		
4	Balducci, 2019*	+	+	+	+	+	+	+	+	+	+	+	+	-	-	+	-	-	+
5	Biddle 2015	+	+	+	+	+	+	?	?	?	+	+	+	+	+	+	?	?	?
6	Butler 2018	?	?	?	-	-	-	+	+	+	?	+	+	+	+	?	-	-	-
7	Carr 2013	+		+	+		+	?		?	+		+	?		+	?		?
8	Danquah 2017	+			+			+			+			+			+		
9	Dunning, 2018	+	+	+	?	?	?	+	+	+	?	?	?	+	+	+	?	?	?
10	Garland, 2018	-			+			-			-			+			-		
11	Graves 2015	+	+	+	+	+	+	+	+	+	+	+	+	?	?	?	?	?	?
12	Healy 2013	-	-	-	+	+	+	+	+	+	+	+	+	+	+	+	-	-	-
13	Healy 2017	+	+	+	+	+	+	?	?	?	+	+	+	+	+	+	?	?	?
14	Kallings 2009	?	?	?	+	+	+	+	+	+	+	+	+	+	+	+	?	?	?
15	Kozey Keadle 2014	-	-	-	-	-	-	?	?	?	+	+	+	+	+	+	-	-	-
16	Lin 2017	?	?	?	+	+	+	?	?	?	+	+	+	+	+	+	?	?	?
17	Lyons 2017	+			+			+			+			+			+		
18	MacEwen 2017	+	+	+	-	-	-	?	?	?	+	+	+	+	+	+	-	-	-
19	Mainsbridge 2014	?			+			+			?			?			?		
20	Malaeb, 2019	-			-			-			?			-			-		
21	Mantzari, 2018	?	?	?	?	?	?	+	+	+	+	+	+	+	+	+	?	?	?
22	Maxwell-Smith 2019	?			+			+			+			+			?		
23	Maylor, 2018	+		+	?		?	+		+	?		?	+		+	?		?
24	Miyamoto 2017	+	+	+	+	+	+	+	+	+	+	+	+	+	+	+	+	+	+
25	Pesola 2017	+	+	+	+	+	+	+	+	+	+	+	+	+	+	+	+	+	+
26	Peterman, 2019	?	?	?	+	+	+	-	-	-	?	+	+	-	-	-	-	-	-
27	Puig-Ribera 2015	?			+			?			+			+			?		
28	Resendiz, 2019	?			?			+			?			+			?		
29	Schuna 2014	?			+			+			+			+			?		
30	Taylor 2016	?	?	?	?	?	?	?	?	?	?	?	?	+	+	+	?	?	?
31	Thomsen 2016	+	+	+	+	+	+	+	+	+	+	+	+	+	+	+	+	+	+
32	Thomsen 2017	+	+	+	+	+	+	+	+	+	+	+	+	+	+	+	+	+	+
33	Zhu, 2018	-	-	-	+	+	+	-	-	-	?	+	+	+	+	+	-	-	-
Total	Low Risk (+)	15	11	13	23	15	16	18	11	12	23	19	20	27	18	20	6	4	5
Total	Unclear risk (?)	12	6	6	6	3	4	11	7	8	9	2	3	3	1	2	17	9	11
Total	High risk (-)	6	4	4	4	3	3	4	3	3	1	0	0	3	2	1	10	8	7
Total	All	33	21	23	33	21	23	33	21	23	33	21	23	33	21	23	33	21	23

Notes: 1=anthropometry & blood pressure outcomes; 2=glucose metabolism outcomes; 3=lipid metabolism outcomes; + = low risk of bias; ? = unclear risk of bias (some concerns); – = high risk of bias.

* Data were extracted from the earlier paper related to this study (Balducci 2017) when it was not reported in the 2019 paper (%BF; FFM; BMI; fasting insulin; HOMA). Risk of bias for Balducci 2017 was low for all criteria.
