# Supplementary material for: Effects of sedentary behaviour interventions on biomarkers of cardiometabolic risk in adults: systematic review with meta-analyses
Source: Br J Sports Med. 2020 Apr 8;55(3):144–54. doi: 10.1136/bjsports-2019-101154 (PMC7841485; doi:10.1136/bjsports-2019-101154)
Supplement: Supplementary data [file bjsports-2019-101154supp013.pdf]

Table S8: Meta-regression: associations of study characteristics with intervention effects on cardiometabolic biomarkers displaying low heterogeneity

	Unadjusted				Age adjusted			
	**k, N**	**b (95% CI)**	**p**	**I^2, p**	**n, N**	**b (95% CI)**	**p**	**I^2, p**
**Weight (kg)**	**25, 1839**			**23.6%, p=0.142**	**25, 1839**			**23.6%, p=0.142**
Mean baseline age, per 10 y	25, 1839	-0.51 (-0.84, -0.18)	0.002	0.0%, p = 0.504		-	-	-
Mean baseline BMI, kg/m^2	25, 1839	-0.02 (-0.15, 0.10)	0.731	26.6%, p = 0.115	25, 1839	0.03 (-0.07, 0.12)	0.579	0.0%, p = 0.462
Mean baseline level	25, 1839	0.00 (-0.04, 0.05)	0.873	26.8%, p = 0.113	25, 1839	0.02 (-0.01, 0.05)	0.313	0.0%, p = 0.505
Sedentary effectiveness, h/day	25, 1839	-0.17 (-0.84, 0.50)	0.625	26.6%, p = 0.115	25, 1839	-0.14 (-0.61, 0.33)	0.565	0.0%, p = 0.463
Duration (vs ≤3 months)	25, 1839		*0.036*	28.0%, p = 0.106	25, 1839		*0.009*	2.4%, p = 0.428
3–6 months		-1.26 (-2.25, -0.27)	0.012			-0.60 (-1.47, 0.26)	0.173	
>6 months		-0.00 (-1.15, 1.15)	0.997			0.25 (-0.66, 1.15)	0.591	
Risk of bias (vs High risk)	25, 1839		*0.640*	29.1%, p = 0.096	25, 1839		*0.020*	5.6%, p = 0.385
Some concerns		-0.47 (-1.85, 0.91)	0.507			0.67 (-0.69, 2.03)	0.335	
Low risk		-0.43 (-1.37, 0.51)	0.366			0.17 (-0.66, 1.00)	0.692	
**Body fat percentage, %**	**16, 1618**			**5.5%, p=0.390**	**16, 1618**			**5.5%, p=0.390**
Mean baseline age, per 10 y	16, 1618	-0.13 (-0.38, 0.13)	0.325	6.5%, p = 0.380		-	-	-
Mean baseline BMI, kg/m^2	16, 1618	-0.04 (-0.13, 0.05)	0.397	8.5%, p = 0.358	16, 1618	-0.02 (-0.14, 0.10)	0.732	13.0%, p = 0.311
Mean baseline level	16, 1618	-0.04 (-0.10, 0.01)	0.130	0.0%, p = 0.482	16, 1618	-0.04 (-0.11, 0.03)	0.238	3.9%, p = 0.408
Sedentary effectiveness, h/day	16, 1618	-0.21 (-0.75, 0.34)	0.456	8.9%, p = 0.354	16, 1618	-0.31 (-0.88, 0.25)	0.274	5.8%, p = 0.388
Duration (vs ≤3 months)	16, 1618		*0.194*	16.2%, p = 0.276	16, 1618		*0.257*	12.1%, p = 0.323
3–6 months		-0.56 (-1.19, 0.06)	0.078			-0.48 (-1.11, 0.16)	0.143	
>6 months		-0.06 (-0.77, 0.66)	0.880			0.11 (-0.67, 0.89)	0.786	
Risk of bias (vs High risk)	16, 1618		*0.209*	16.3%, p = 0.276	16, 1618		*0.332*	17.5%, p = 0.267
Some concerns		-0.76 (-1.61, 0.09)	0.081			-0.68 (-1.57, 0.21)	0.132	
Low risk		-0.51 (-1.38, 0.35)	0.243			-0.44 (-1.34, 0.45)	0.331	
**Fat mass, kg**	**6, 724**			**26.6%, p=0.235**	**6, 724**			**26.6%, p=0.235**
Mean baseline age, per 10 y	6, 724	-0.22 (-0.64, 0.19)	0.296	26.7%, p = 0.243		-	-	-
Mean baseline BMI, kg/m^2	6, 724	-0.12 (-0.30, 0.06)	0.179	17.1%, p = 0.306	6, 724	-0.10 (-0.34, 0.14)	0.416	35.4%, p = 0.200

Hadgraft NT, *et al. Br J Sports Med* 2021; 55:144–156. doi: 10.1136/bjsports-2019-101154

Mean baseline level	6, 724	-0.08 (-0.18, 0.01)	0.082	0.0%, p = 0.436	6, 724	-0.09 (-0.22, 0.05)	0.212	20.6%, p = 0.286
Sedentary effectiveness, h/day	6, 724	-0.67 (-1.71, 0.36)	0.202	22.8%, p = 0.269	6, 724	-0.78 (-1.72, 0.16)	0.104	0.0%, p = 0.422
Duration (vs ≤3 months)	6, 724		*0.506*	55.6%, p = 0.080	6, 724		*0.685*	63.1%, p = 0.067
3–6 months		-0.83 (-2.24, 0.58)	0.247			-1.61 (-5.37, 2.16)	0.403	
>6 months		-0.06 (-1.45, 1.33)	0.930			-0.20 (-1.74, 1.35)	0.804	
Risk of bias (vs High risk)	6, 724		*0.637*	50.3%, p = 0.110	6, 724		*0.837*	63.1%, p = 0.066
Some concerns		-0.30 (-1.48, 0.89)	0.624			-0.22 (-1.75, 1.31)	0.778	
Low risk		-0.56 (-1.73, 0.60)	0.344			-0.37 (-2.37, 1.63)	0.716	
**Systolic Blood Pressure, mmHg**	**25, 1932**			**8.6%, p=0.341**	**25, 1932**			**8.6%, p=0.341**
Mean baseline age, per 10 y	25, 1932	0.38 (-0.49, 1.24)	0.392	8.1%, p = 0.349		-	-	-
Mean baseline BMI, kg/m^2	24, 1903	0.02 (-0.29, 0.34)	0.882	15.1%, p = 0.256	24, 1903	-0.02 (-0.35, 0.32)	0.922	15.7%, p = 0.251
Mean baseline level	24, 1911	0.04 (-0.11, 0.18)	0.617	4.3%, p = 0.402	24, 1911	0.12 (-0.11, 0.36)	0.303	4.8%, p = 0.396
Sedentary effectiveness, h/day	23, 1882	-0.48 (-2.16, 1.20)	0.573	7.9%, p = 0.355	23, 1882	-0.71 (-2.64, 1.22)	0.472	11.3%, p = 0.312
Duration (vs ≤3 months)	25, 1932		*0.708*	14.7%, p = 0.261	25, 1932		*0.743*	14.0%, p = 0.273
3–6 months		0.31 (-2.49, 3.10)	0.828			0.16 (-2.67, 2.99)	0.912	
>6 months		-1.09 (-4.01, 1.84)	0.466			-1.19 (-4.13, 1.76)	0.430	
Risk of bias (vs High risk)	25, 1932		*0.375*	10.8%, p = 0.313	25, 1932		*0.607*	12.9%, p = 0.288
Some concerns		1.54 (-1.57, 4.65)	0.332			1.40 (-2.10, 4.90)	0.433	
Low risk		1.80 (-0.76, 4.36)	0.168			1.66 (-1.21, 4.53)	0.258	
**HDL Cholesterol, mM**	**22, 1760**			**22.5%, p=0.168**	**22, 1760**			**22.5%, p=0.168**
Mean baseline age, per 10 y	22, 1760	-0.01 (-0.03, 0.00)	0.011	0.7%, p = 0.450		-	-	-
Mean baseline BMI, kg/m^2	22, 1760	0.00 (-0.01, 0.00)	0.304	14.3%, p = 0.273	22, 1760	-0.00 (-0.01, 0.01)	0.768	5.2%, p = 0.392
Mean baseline level	21, 1739	-0.08 (-0.23, 0.08)	0.344	6.3%, p = 0.378	21, 1739	-0.06 (-0.22, 0.09)	0.428	7.4%, p = 0.365
Sedentary effectiveness, h/day	21, 1739	-0.02 (-0.06, 0.01)	0.222	3.3%, p = 0.416	21, 1739	-0.04 (-0.08, 0.00)	0.046	0.0%, p = 0.583
Duration (vs ≤3 months)	22, 1760		*0.266*	27.6%, p = 0.124	22, 1760		*0.163*	10.6%, p = 0.325
3–6 months		-0.05 (-0.12, 0.01)	0.104			-0.04 (-0.10, 0.02)	0.175	
>6 months		-0.02 (-0.08, 0.05)	0.604			-0.01 (-0.07, 0.05)	0.735	
Risk of bias (vs High risk)	22, 1760		*0.023*	9.6%, p = 0.335	22, 1760		*0.035*	5.2%, p = 0.392

Some concerns		-0.06 (-0.12, 0.00)	0.035			-0.09 (-0.18, 0.01)	0.076	
Low risk		-0.06 (-0.10, -0.01)	0.015			-0.07 (-0.14, 0.00)	0.043	

Table presents unstandardized regression coefficient (*b*) and 95% confidence interval (CI) and *p* value from meta-regression of controlled trials of adult sedentary behaviour interventions ≥7 days. Italics indicates overall p value (omnibus test). No meta-regression performed for BMI or LDL cholesterol, since I^2=0.

[a] k = total number of interventions included and n = total number of individuals analysed in the included interventions, in the meta-regressions or main metaanalysis (boldface) [b] Residual heterogeneity (I^2 and p from Cochrane's Q test) with overall heterogeneity in the main metaanalysis shown in boldface

Hadgraft NT, *et al. Br J Sports Med* 2021; 55:144–156. doi: 10.1136/bjsports-2019-101154
