# Supplementary figures and images for: Effects of sedentary behaviour interventions on biomarkers of cardiometabolic risk in adults: systematic review with meta-analyses
Source: Br J Sports Med. 2020 Apr 8;55(3):144–54. doi: 10.1136/bjsports-2019-101154 (PMC7841485; doi:10.1136/bjsports-2019-101154)

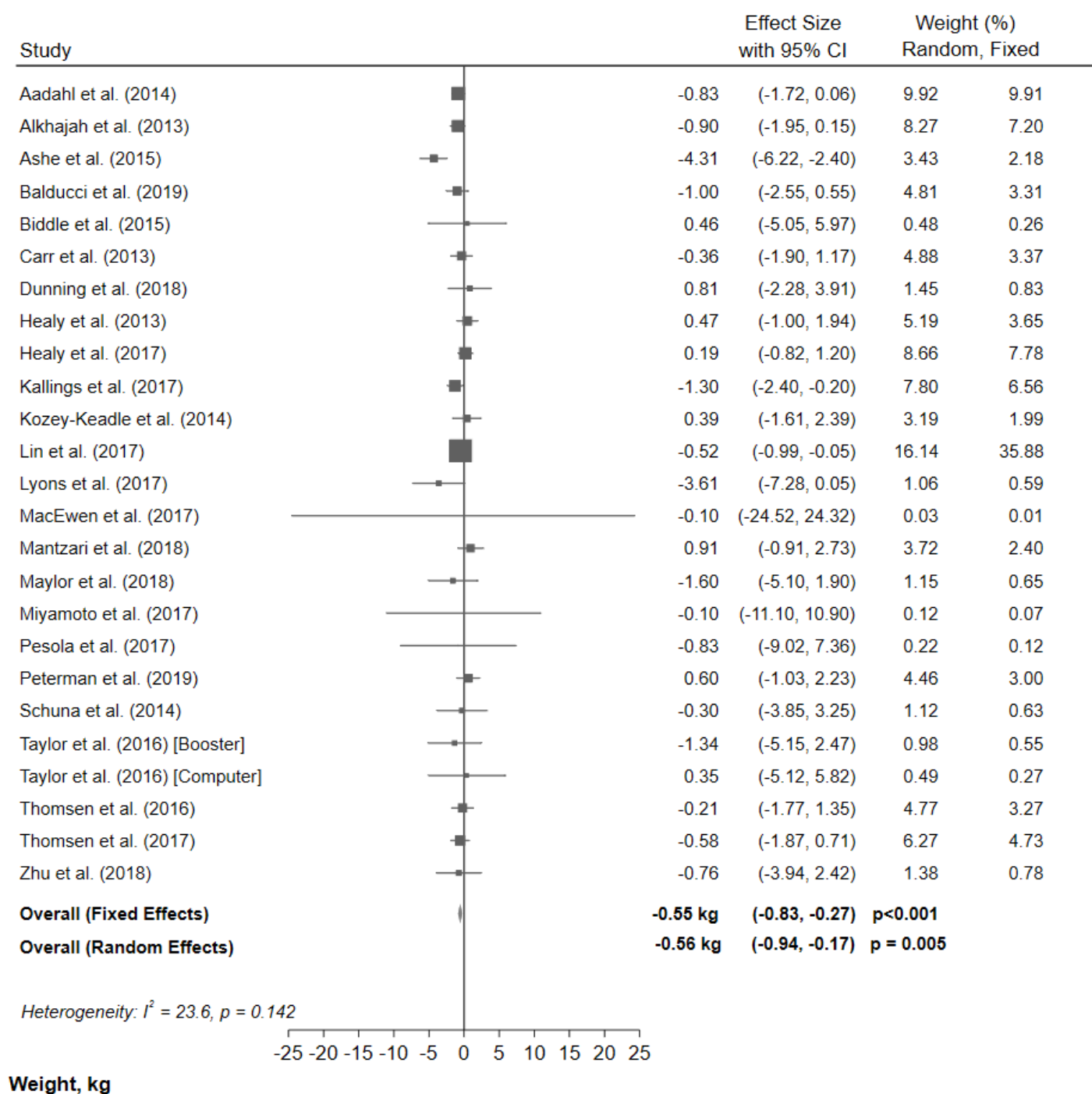

Supplement: Supplementary data [file bjsports-2019-101154supp007.pdf]

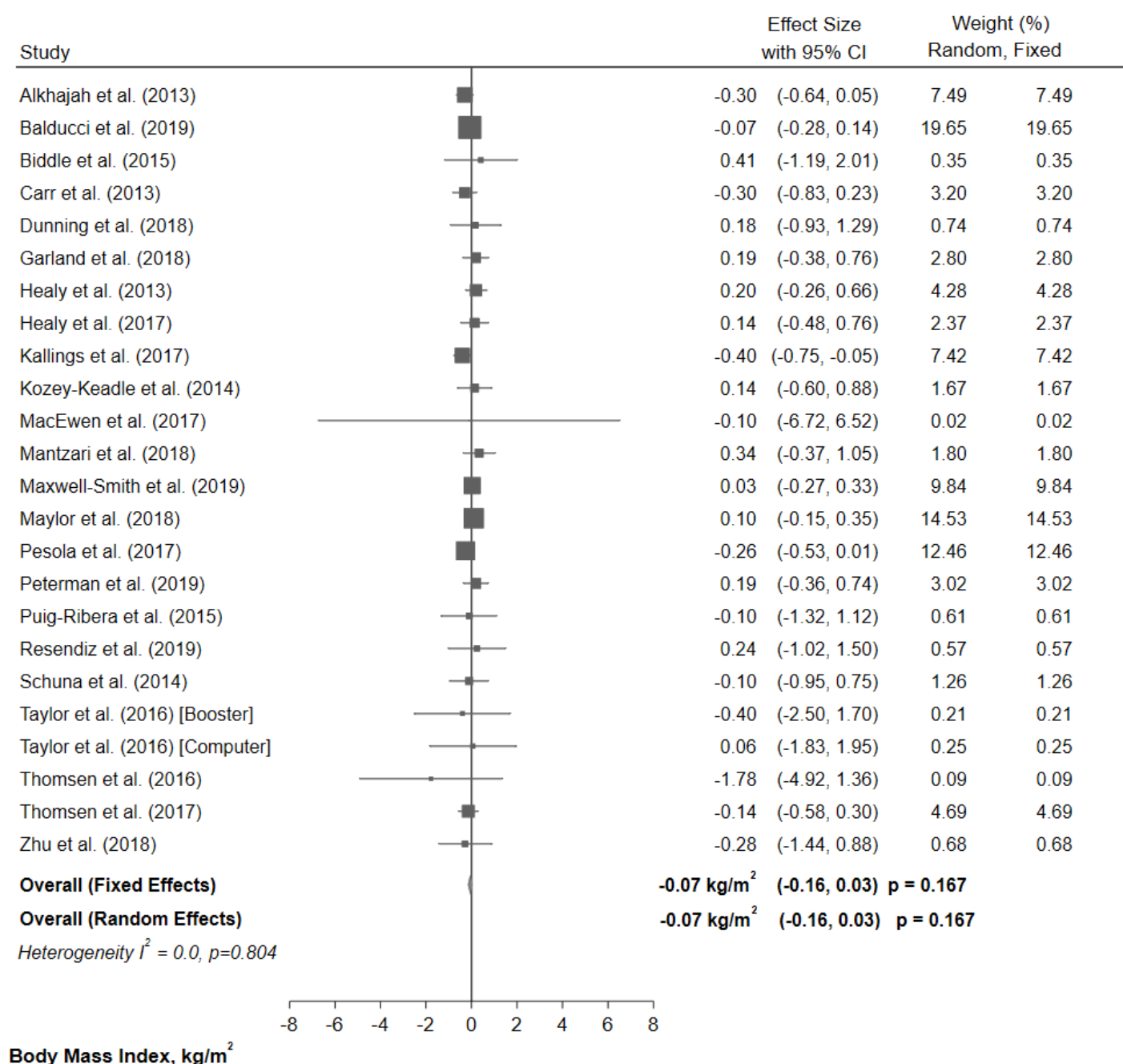

Supplement: Supplementary data [file bjsports-2019-101154supp008.pdf]

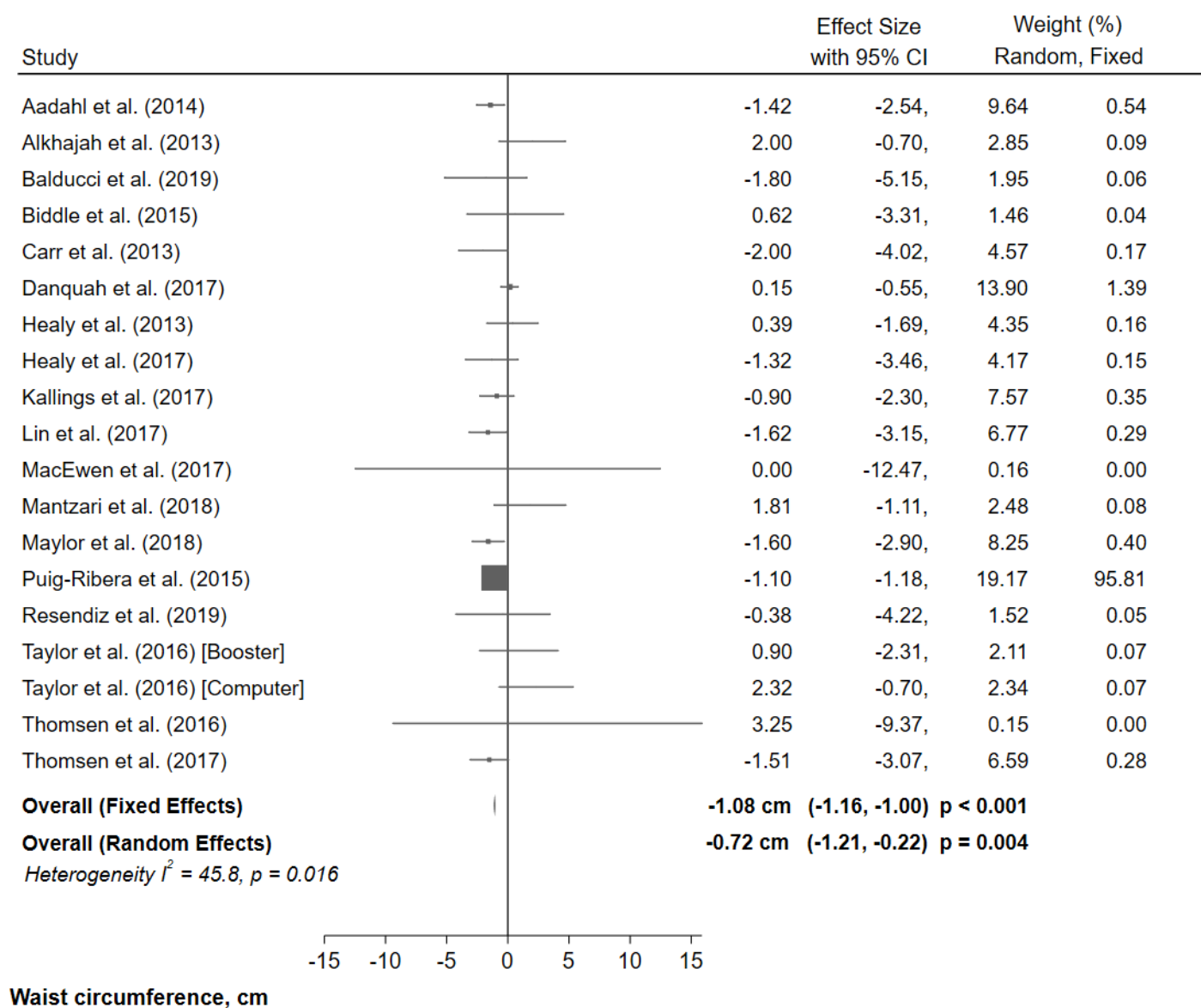

Supplement: Supplementary data [file bjsports-2019-101154supp009.pdf]

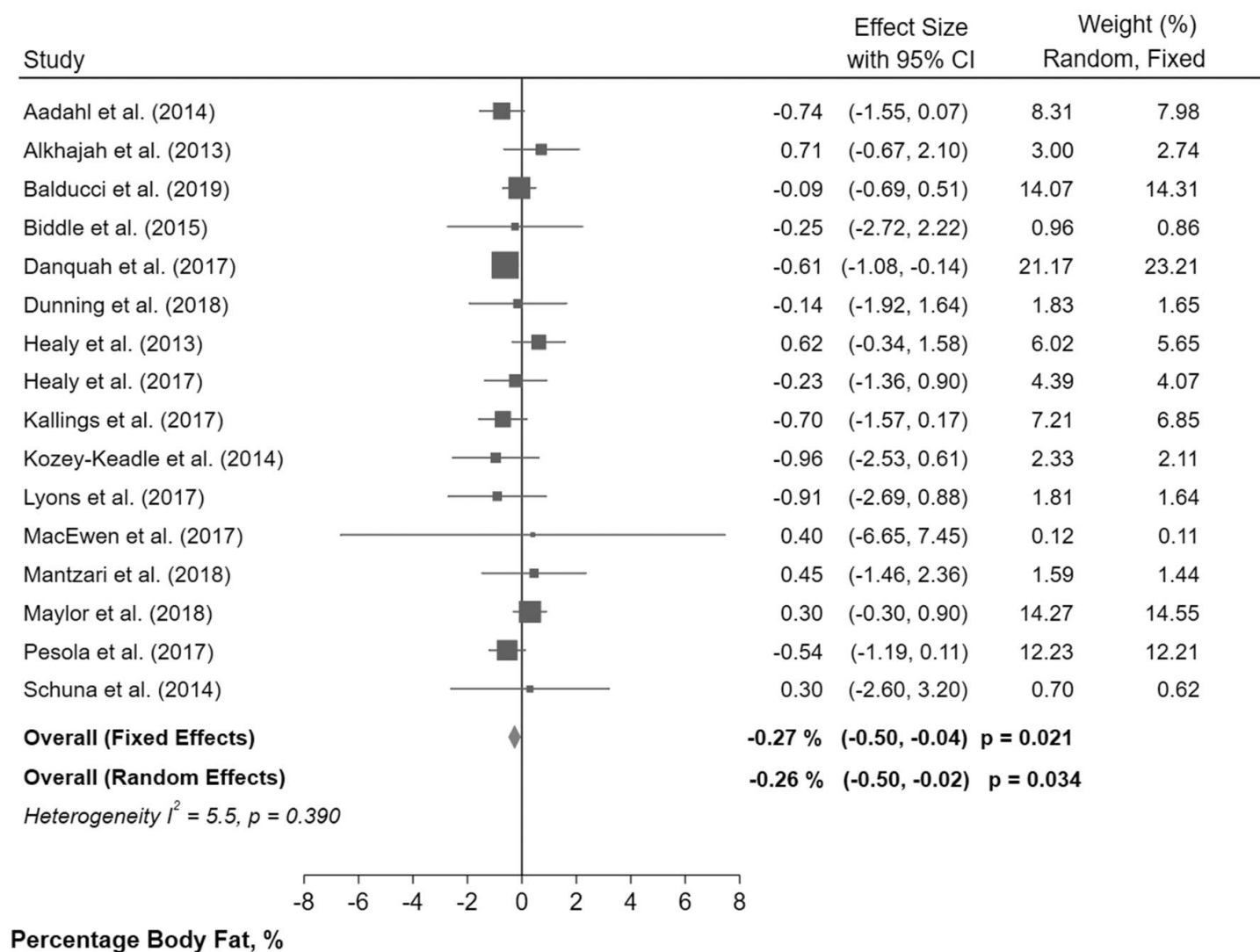

Supplement: Supplementary data [file bjsports-2019-101154supp010.pdf]

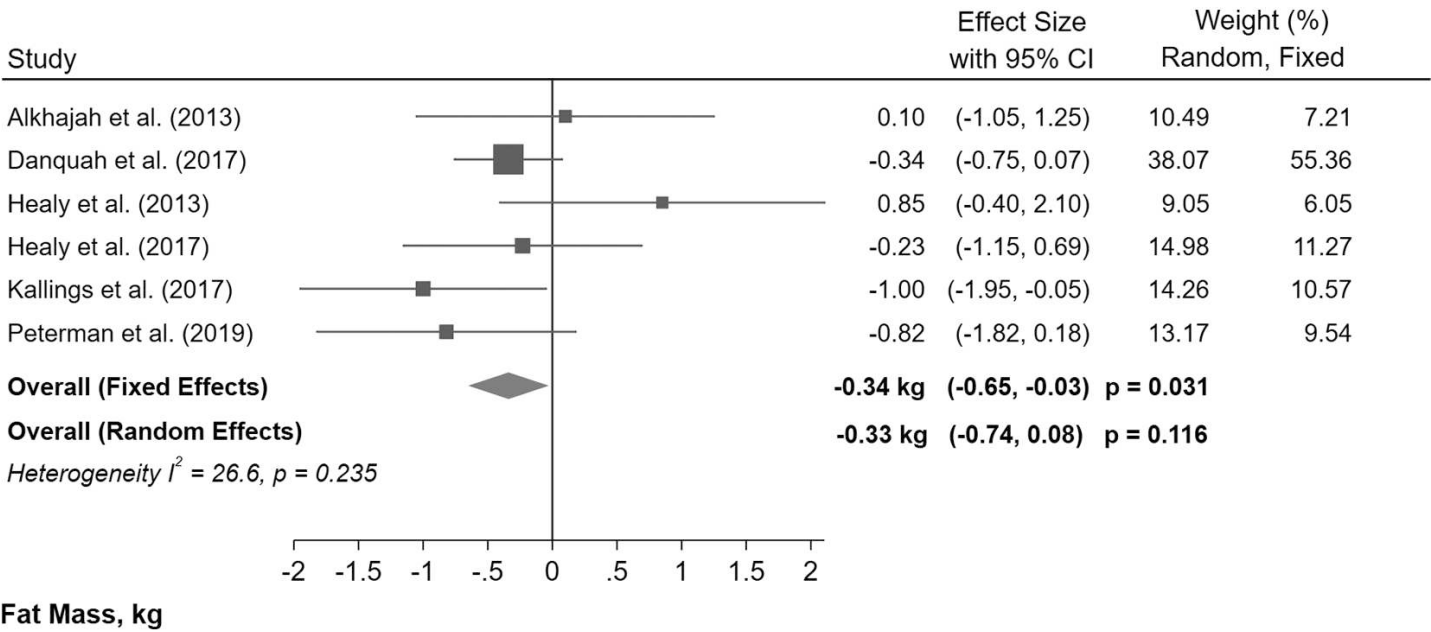

Supplement: Supplementary data [file bjsports-2019-101154supp011.pdf]

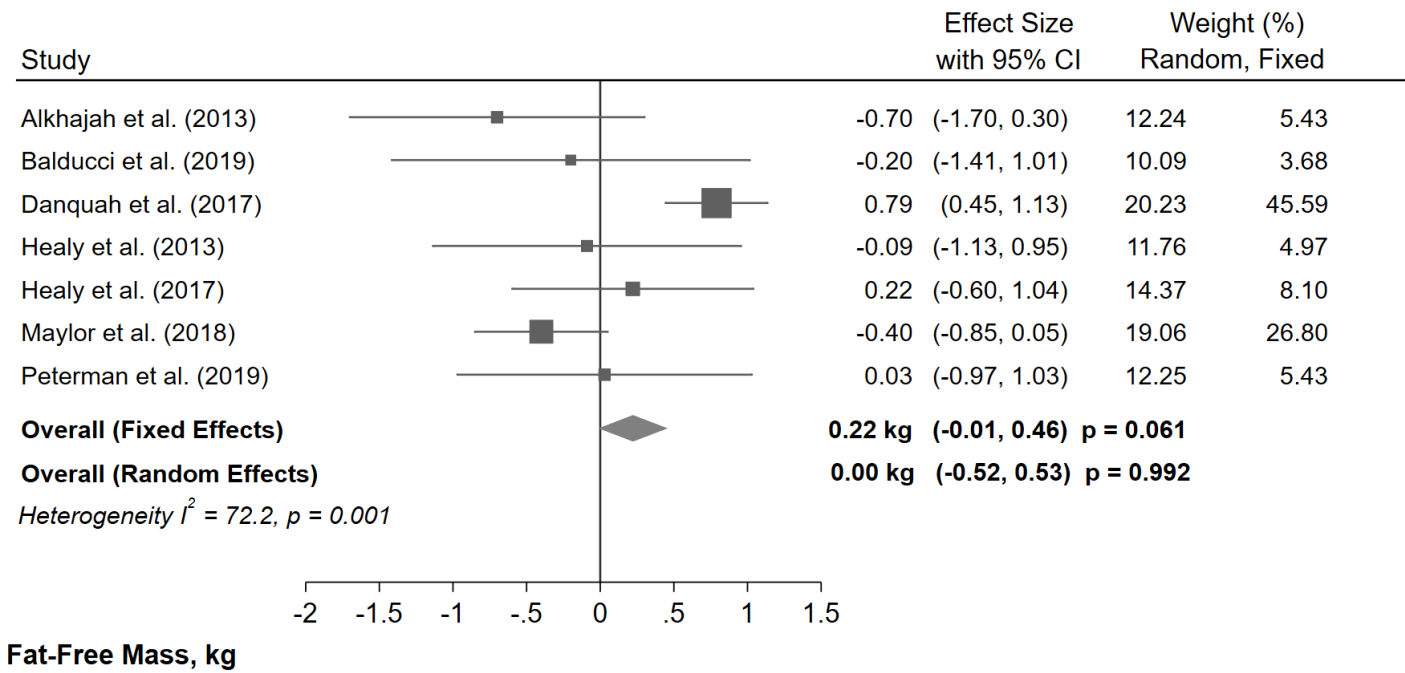

Supplement: Supplementary data [file bjsports-2019-101154supp012.pdf]

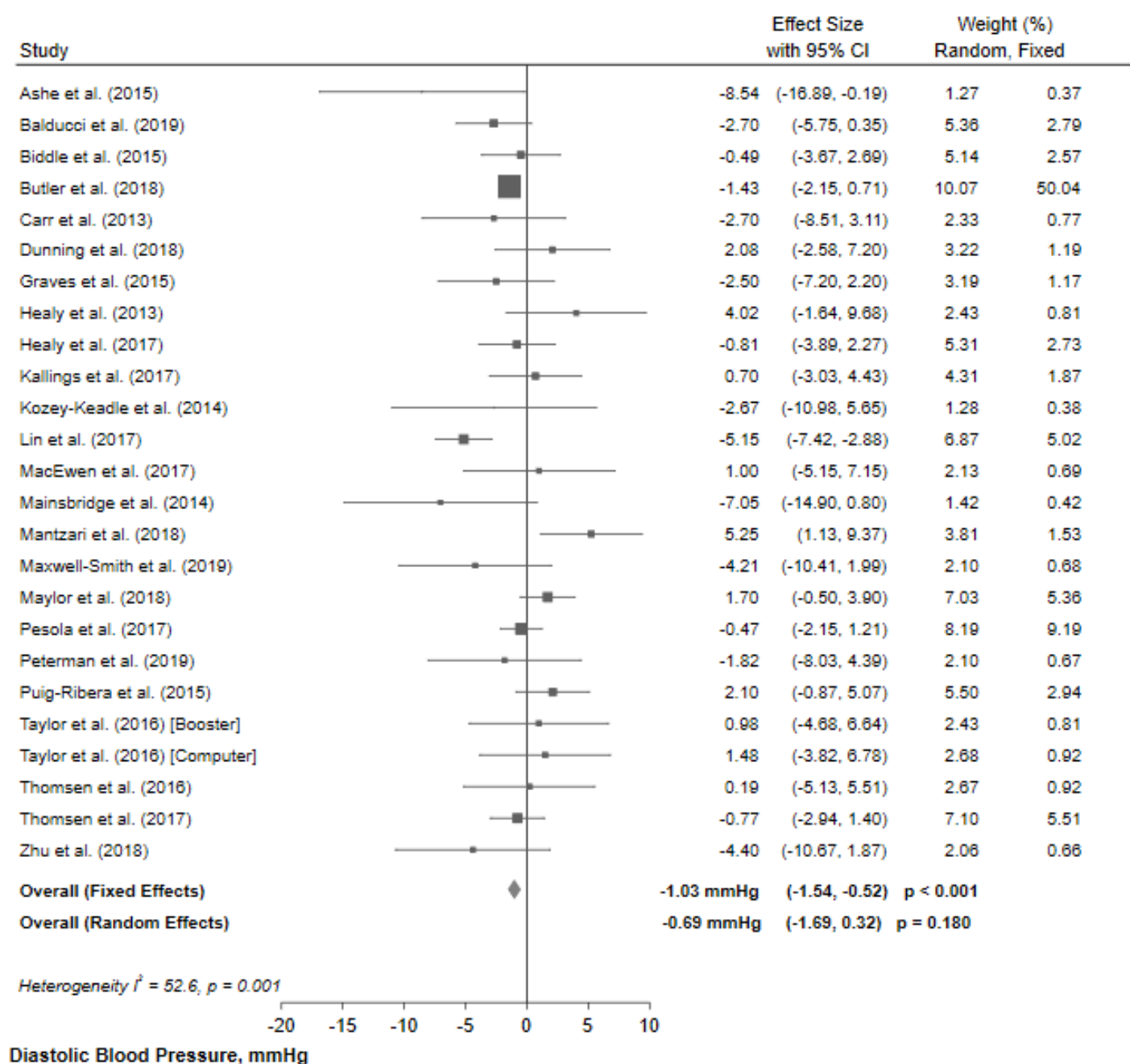

Supplement: Supplementary data [file bjsports-2019-101154supp014.pdf]

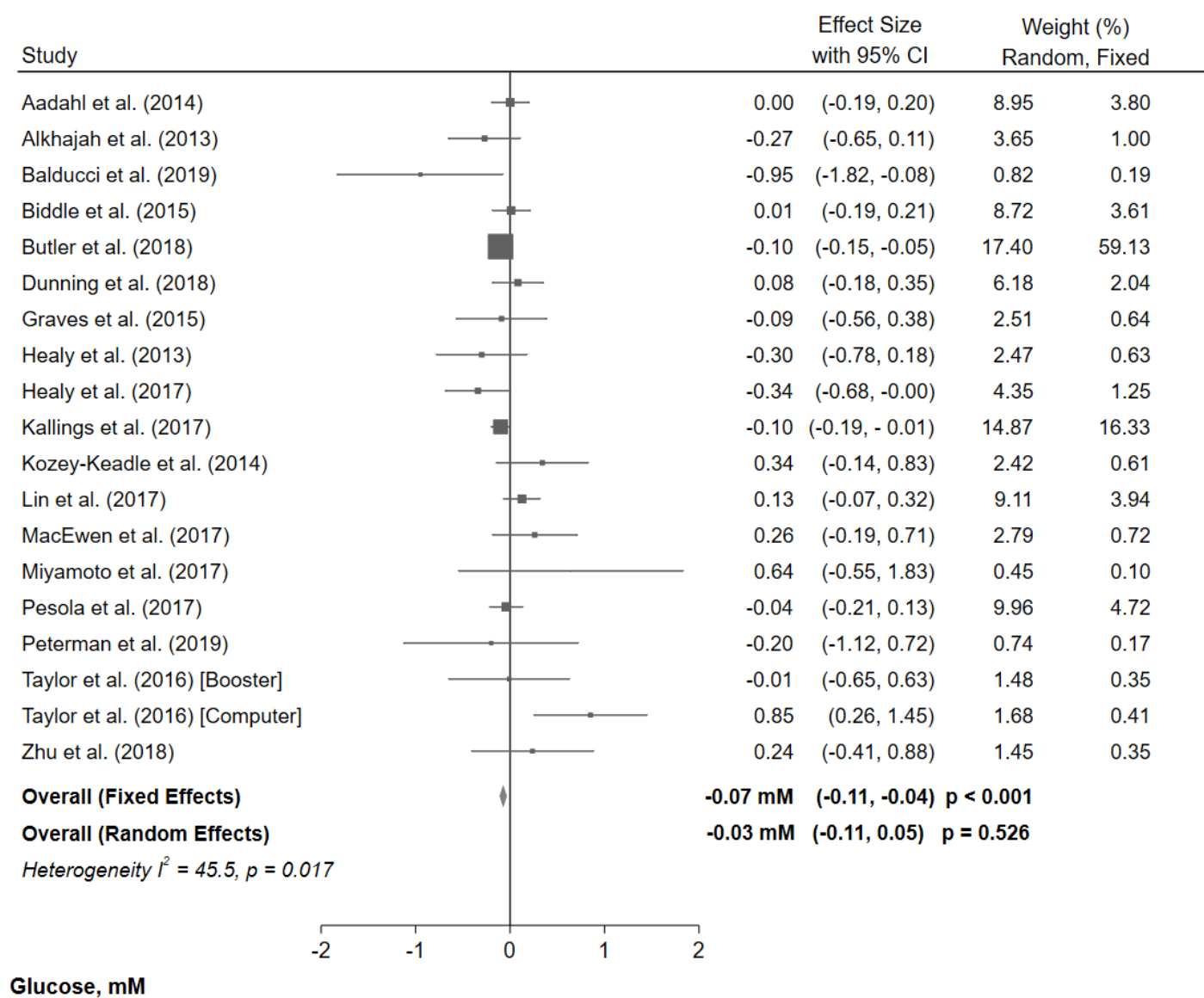

Supplement: Supplementary data [file bjsports-2019-101154supp016.pdf]

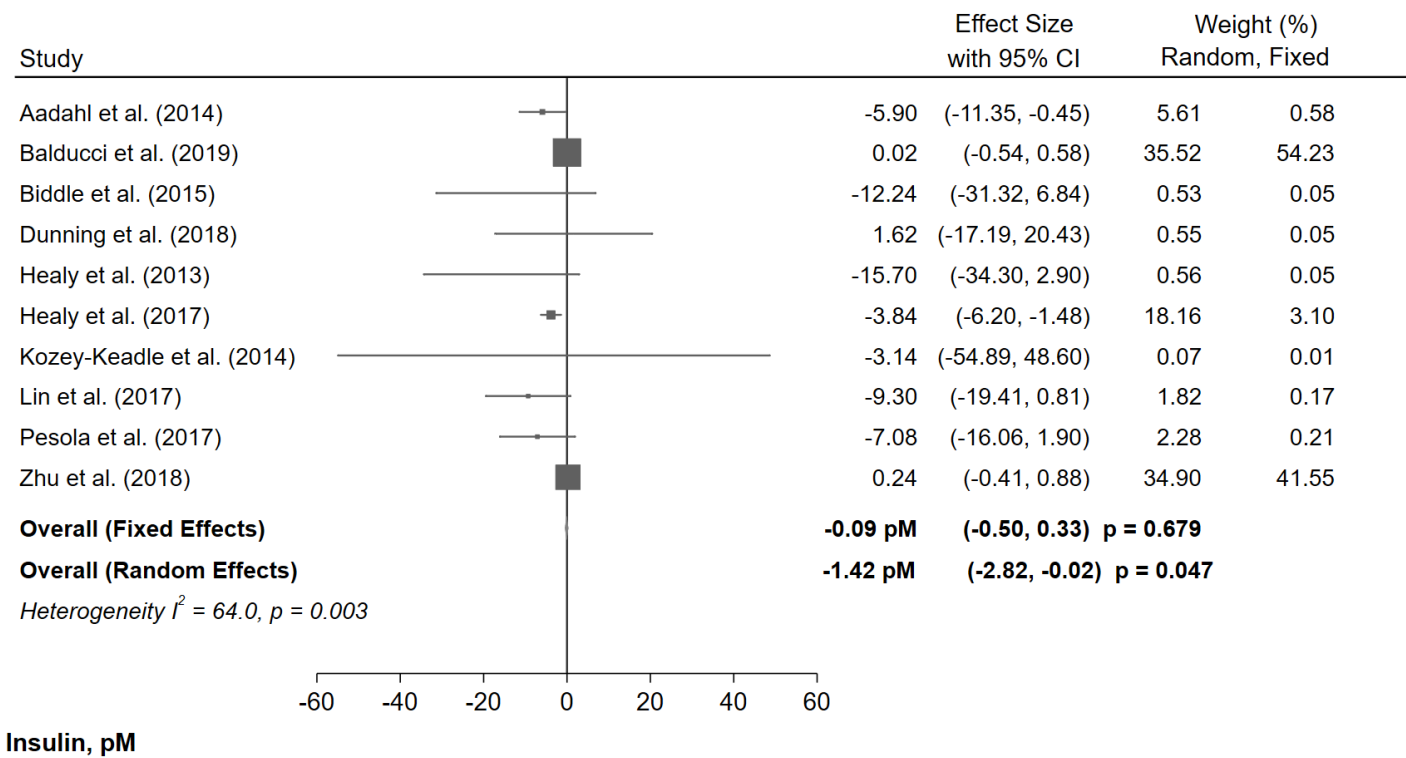

Supplement: Supplementary data [file bjsports-2019-101154supp017.pdf]

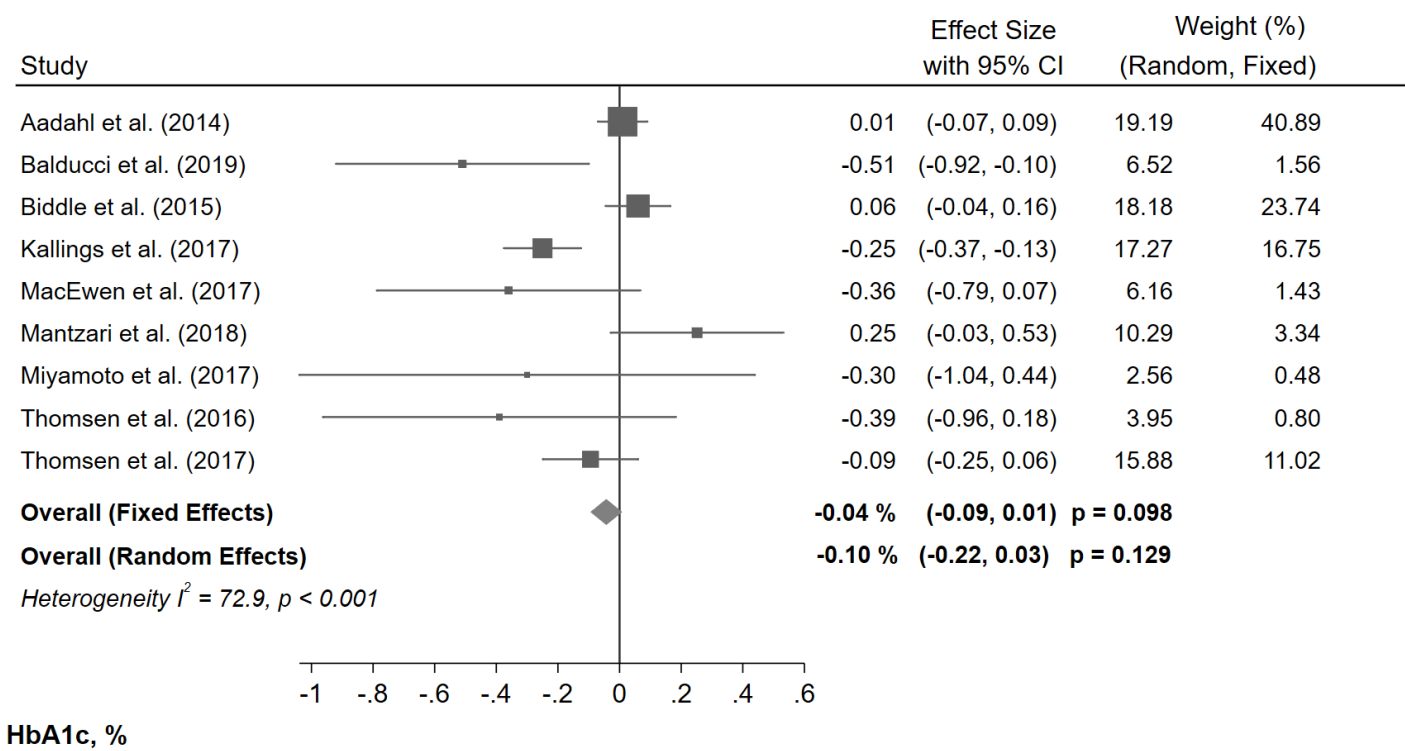

Supplement: Supplementary data [file bjsports-2019-101154supp018.pdf]

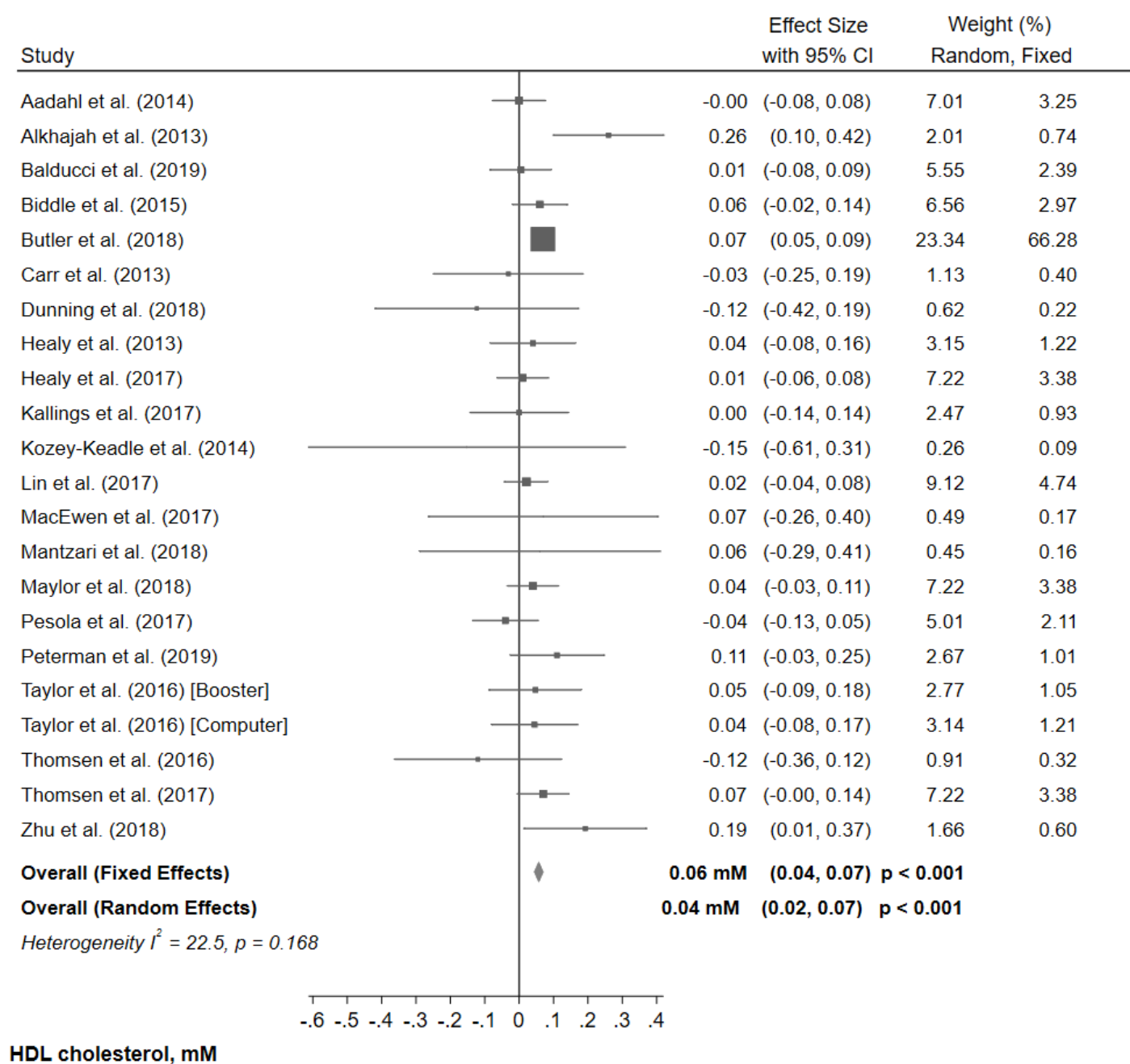

Supplement: Supplementary data [file bjsports-2019-101154supp019.pdf]

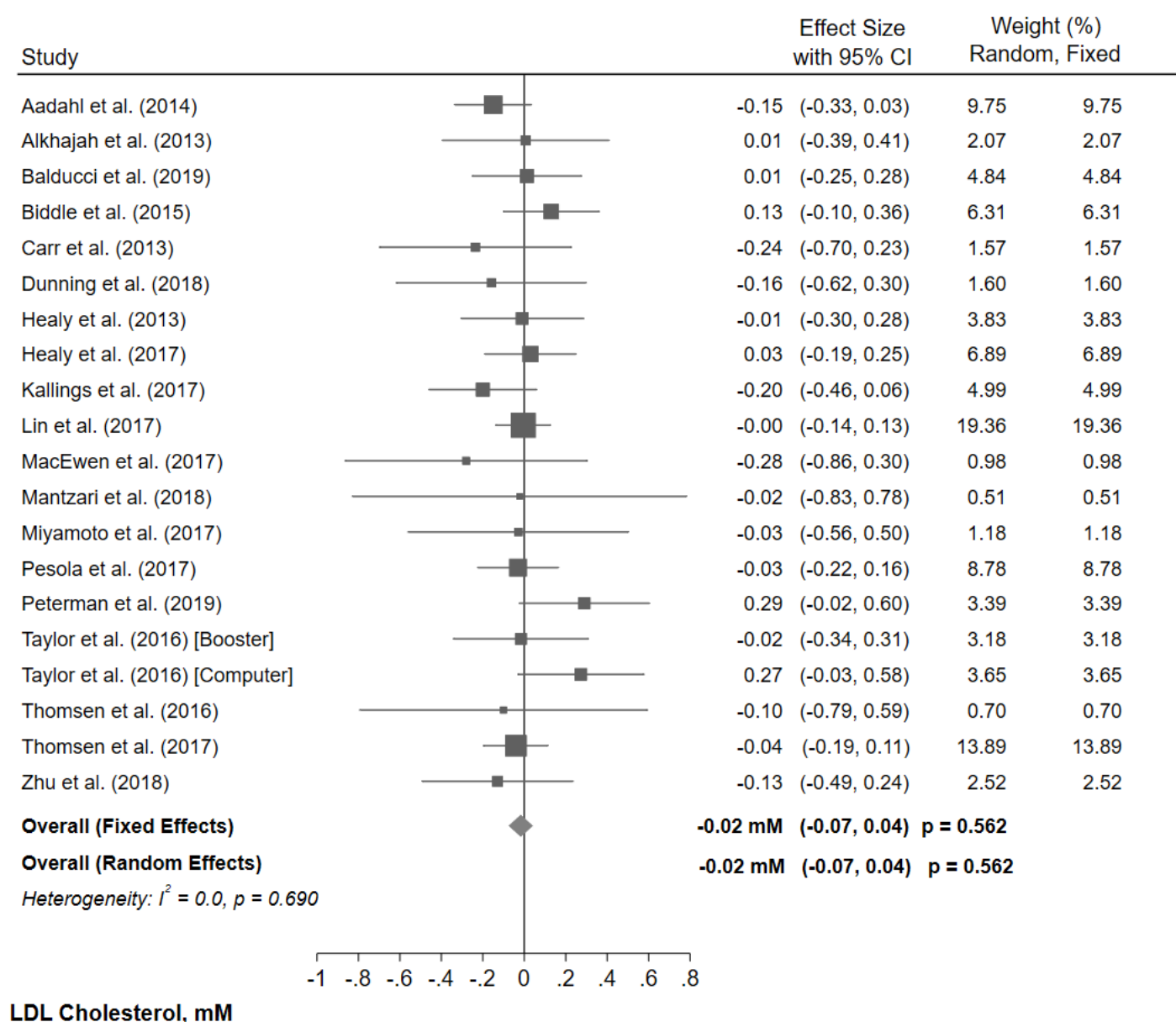

Supplement: Supplementary data [file bjsports-2019-101154supp020.pdf]

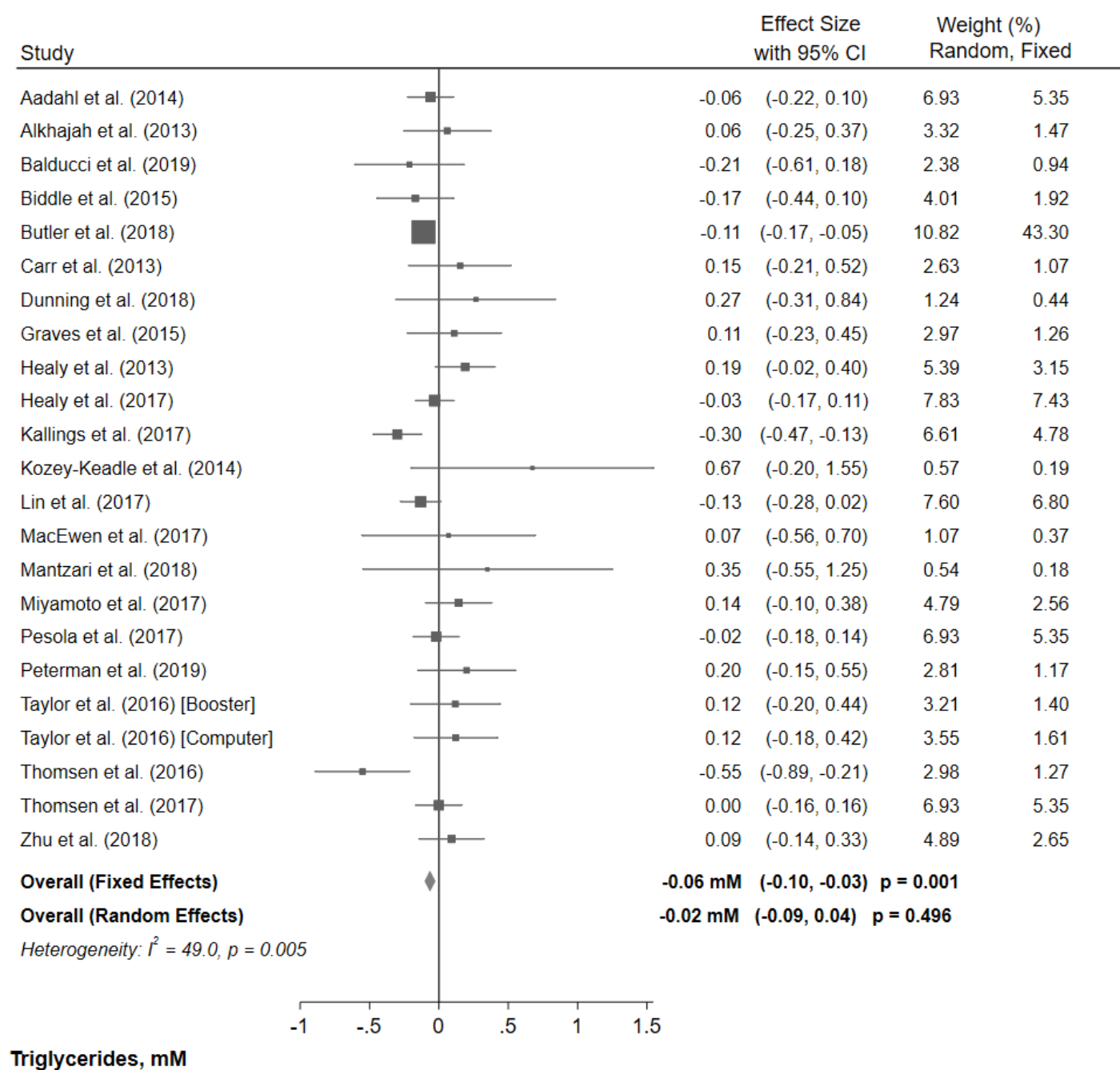

Supplement: Supplementary data [file bjsports-2019-101154supp021.pdf]
