# Supplementary material for: Effects of sedentary behaviour interventions on biomarkers of cardiometabolic risk in adults: systematic review with meta-analyses
Source: Br J Sports Med. 2020 Apr 8;55(3):144–54. doi: 10.1136/bjsports-2019-101154 (PMC7841485; doi:10.1136/bjsports-2019-101154)
Supplement: Supplementary data [file bjsports-2019-101154supp015.pdf]

Supplementary Table S5 Study baseline levels of blood biomarkers (mean ± SD) in controlled trials of 34 adult sedentary behaviour interventions ≥7 days

**Study**	**TC**	**HDL**	**LDL**	**TG**	**Glucose**	**Insulin**	**HbA1c**
	mM	***mM***	***mM***	***mM***	***mM***	***pmol/L***	***%***
Aadahl et al. (2014)	5.3 ± 1.0	1.5 ± 1.5	3.2 ± 3	1.2 ± 1.1	5.6 ± 1.0	52.1 ± 45	5.6 ± 0.5
Alkhajah et al. (2012)	4.5 ± 0.9	1.5 ± 0.4	2.7 ± 0.7	0.9 ± 0.8	4.9 ± 0.4		
Ashe et al. (2015)							
Balducci et al. (2019)	4.7 ± 1.0	1.2 ± 0.4	2.9 ± 0.9	1.5 ± 0.9 [a]	7.6 ± 2.7	77.4 ± 74.7 [a]	7.4 ± 1.5
Biddle et al. (2015)	4.9 ± 1.0	1.3 ± 0.3	3.0 ± 0.7	1.5 ± 1.2	4.8 ± 0.6	86 ± 56.5	5.6 ± 0.3
Butler (2018)							5.4 ± 2.5
Carr et al. (2013)	4.9 ± 0.7	1.2 ± 0.5	3.0 ± 0.7	1.3 ± 0.6			
Danquah et al. (2017)							
Dunning (2018)	4.8 ± 0.8	1.6 ± 0.3	2.8 ± 0.7	1.0 ± 0.5	4.7 ± 0.4	37.1 ± 15.2	
Garland (2018)							
Graves et al. (2015)	4.2 ± 0.9			1.6 ± 0.7	5.1 ± 0.7		
Healy et al. (2013)	5 ± 0.9	1.5 ± 0.4	2.9 ± 0.8	1.1 ± 0.5	4.8 ± 0.9	52.7 ± 28.9	
Healy et al. (2017)	5.4 ± 1.0	1.5 ± 0.4	3.2 ± 0.9	1.4 ± 0.7	5.1 ± 1.4	61.4 ± 112	
Kallings et al. (2009)	5.5 ± 0.1	1.7 ± 0.1	3.3 ± 0.1	1.3 ± 0.1	5.4 ± 0.1		4.9 ± 0.1
Kozey-Keadle et al. (2014)	4.7 ± 0.8	1.7 ± 0.5		1.9 ± 1.2	5.8 ± 0.7	133 ± 86.6	
Lin et al. (2017)	5 ± 0.9	1.5 ± 0.4	2.8 ± 0.7	1.3 ± 0.7	4.8 ± 0.7	42.6 ± 31.2	
Lyons et al. (2017)							
MacEwen et al. (2017)	5.1 ± 0.9	1.5 ± 0.4	2.9 ± 0.7	1.6 ± 0.8	5.8 ± 0.5		6.9 ± 0.5
Mainsbridge et al. (2014)							
Malaeb et al. (2019)							
Mantzari et al. (2018)	4.8 ± 1.0	1.4 ± 0.3	2.7 ± 1.0	1.4 ± 0.6			5.2 ± 2.4
Maxwell-Smith et al. (2018)							
Maylor et al. (2018)	4.4 ± 0.4	1.4 ± 0.2					
Miyamoto et al. (2017)	5.3 ± 0.2		3.3 ± 0.2	1.2 ± 0.1	7.0 ± 0.4		7.0 ± 0.2
Pesola et al. (2017)	4.8 ± 0.8	1.8 ± 0.5	2.6 ± 0.9	1.0 ± 0.9	5.3 ± 0.5	38.2 ± 25.6	
Peterman et al. (2019)	4 ± 0.8	1.1 ± 0.2	2.5 ± 0.7	1.2 ± 0.6	4.1 ± 0.6		

Puig-Ribera et al. (2015)							
Resendiz et al. (2019)							
Schuna et al. (2014)							
Taylor et al. (2016) - Booster breaks	5.0 ± 0.4	1.4 ± 0.2	2.9 ± 0.3	1.4 ± 0.5	5.4 ± 2.1		
Taylor et al. (2016) - Computer breaks	4.8 ± 0.3	1.3 ± 0.2	2.8 ± 0.3	1.3 ± 0.3	5.3 ± 1.3		
Thomsen et al. (2016)	5.5 ± 1.3	1.5 ± 0.4	3.1 ± 1.1	1.4 ± 0.7			5.2 ± 0.1
Thomsen et al. (2017)	5.2 ± 1.1	1.6 ± 0.5	3.1 ± 0.9	1.2 ± 0.6			5.5 ± 0.8
Zhu et al. (2018)	4.7 ± 0.7	1.6 ± 0.5	2.9 ± 0.7	1.1 ± 0.6	5.4 ± 0.7	73.6 ± 41.4	

TC = Total Cholesterol; HDL = High-density lipoprotein cholesterol; LDL = Low-density lipoprotein cholesterol; TG = triglycerides

[a] Different value reported in relation to the interim outcomes in Balducci 2017: 1.8 ± 1.4 mM triglycerides and 89.5 ± 86.4 pM insulin.
