# Supplementary material for: Effects of sedentary behaviour interventions on biomarkers of cardiometabolic risk in adults: systematic review with meta-analyses
Source: Br J Sports Med. 2020 Apr 8;55(3):144–54. doi: 10.1136/bjsports-2019-101154 (PMC7841485; doi:10.1136/bjsports-2019-101154)
Supplement: Supplementary data [file bjsports-2019-101154supp005.pdf]

Supplementary Table S4 Baseline anthropometric characteristics (mean ± SD) and effects on sedentary behaviour in controlled trials of 34 adult sedentary behaviour interventions ≥7 days

Study	SB effectiveness [a]	Weight	BMI	Waist	Body fat	Fat mass	Fat-free mass	Systolic BP	Diastolic BP
	min/day	*kg*	*kg/m²*	*cm*	*%*	*kg*	*kg*	*mmHg*	*mmHg*
Aadahl et al. (2014)	-19.2	80.4 ± 16.3	27.3 ± 5.0	93.8 ± 13.5	32.5 ± 9.3				
Alkhajah et al. (2012)	-78.0	62.2 ± 9.4	22.1 ± 2.6	81.8 ± 8.8	29.2 ± 4.6	18.4 ± 4.5	44.1 ± 6.2		
Ashe et al. (2015)	-21.1	77.9 ± 19.2	29.3 ± 6.8					133 ± 15	82 ± 8
Balducci et al. (2019)	-48.0 [b]	84.1 ± 16.5	30.1 ± 5.1	103.6 ± 12.8	31.7 ± 10.2		56.5 ± 11.4	140 ± 20	83 ± 12
Biddle et al. (2015)	-7.2	98.6 ± 18.8	34.6 ± 5.1	103.3 ± 14.2	40.6 ± 7.1			120 ± 15	84 ± 10
Butler (2018)		70.6 ± 14.3	23.0 ± 3.0	74.4 ± 6.9					
Carr et al. (2013)	-18.2	89.4 ± 15.0	32.4 ± 4.7	92.7 ± 11.2				119 ± 13	76 ± 10
Danquah et al. (2017)	-27.0		26.0 ± 4.9	91.6 ± 13.0	30.2 ± 7.6	23.9 ± 11.1	53.4 ± 11.1		
Dunning et al. (2018)	-24.0	69.6 ± 9.9	23.8 ± 2.8		24.5 ± 7.9			109 ± 8	74 ± 8
Garland et al. (2018)	-41.1		24.8 ± 4.9						
Graves et al. (2015)	-51.6	68.8 ± 15.0	24.8 ± 4.5					119 ± 13	73 ± 9
Healy et al. (2013)	-82.6	80.2 ± 17.1	26.8 ± 5.4	91.2 ± 13.6	29.4 ± 10.0	24.2 ± 11.3	56.1 ± 11.3	131 ± 14	78 ± 8
Healy et al. (2017)	-36.3	80.2 ± 19.7	28.6 ± 6.1	93.6 ± 14.9	33.1 ± 9.5	27.0 ± 13.0	51.7 ± 11.1	127 ± 15	78 ± 11
Kallings et al. (2009)	-60.0	88.2 ± 12.6	30.1 ± 3.1	105.8 ± 8.5	36.7 ± 7.2	32.3 ± 7.5		140 ± 2	81 ± 1
Kozey-Keadle et al. (2014)	-88.3	99.4 ± 14.8	35.0 ± 4.7		45.5 ± 5.8			130 ± 10	79 ± 9
Lin et al. (2017)	-0.9	64.8 ± 12.1	24.0 ± 3.2	82.8 ± 9.4				118 ± 14	79 ± 10
Lyons et al. (2017)	-27.0	82.4 ± 10.9	30.3 ± 3.5		45.1 ± 5.3				
MacEwen et al. (2017)	-97.4	99.6 ± 24.6	35.8 ± 8.3	111.4 ± 13.6	42.7 ± 8.3			129 ± 12	86 ± 8
Mainsbridge et al. (2014)								135 ± 17	85 ± 14
Malaeb et al. (2019)		78.5 ± ?	28.4 ± ?		38.5 ± ?	28.5 ± ?	45.5 ± ?		
Mantzari et al. (2018)	-59.9	74.5 ± 14.7	25.1 ± 4.8	87.7 ± 13.5	26.8 ± 10.4			128 ± 13	78 ± 9
Maxwell-Smith et al. (2018)	8.6		28.3 ± 5.0					142 ± 18	86 ± 12
Maylor et al. (2018)	-1.0	76.4 ± 7.6	25.9 ± 0.5	86.5 ± 2.8	28.8 ± 1.4			126 ± 7	78 ± 5
Miyamoto et al. (2017)	-77.8	66.6 ± 3.9	24.6 ± 1.1						
Pesola et al. (2017)	11.3	71.9 ± 14.8	24.5 ± 3.8		27.8 ± 8.1			117 ± 11	74 ± 8
Peterman et al. (2019)	-12.1	79.0 ± 17.0	28.3 ± 6.7			28.5 ± 13.0	47.8 ± 8.1	112 ± 10	69 ± 8
Puig-Ribera et al. (2015)	-16.3		25.7 ± 4.4	87.9 ± ?				121 ± 17	78 ± 11

Resendiz et al. (2019)	-95.7		32.2 ± 5.2	96.8 ± 12.2					
Schuna et al. (2014)	-21.6	97.0 ± 24.5	35.9 ± 8.5		45.0 ± 5.6				
Taylor et al. (2016) – Booster breaks	-85.7	87.3 ± 15.1	31.2 ± 3.0	100.8 ± 8.3				117 ± 19	70 ± 8
Taylor et al. (2016) – Computer breaks	-87.7	78.6 ± 12.2	29.0 ± 2.6	96.5 ± 6.8				117 ± 17	70 ± 8
Thomsen et al. (2016)	-27.6	78.4 ± 17.3	25.5 ± 6.5	86.7 ± 18.5				128 ± 21	78 ± 11
Thomsen et al. (2017)	-132	75.2 ± 17.2	26.4 ± 5.4	92.1 ± 14.1				131 ± 20	78 ± 10
Zhu et al. (2018)	8.0	74.4 ± 18.1	25.8 ± 4.9					119 ± 15	76 ± 10

BMI = Body Mass Index; SB = Sedentary Behaviour; BP = Blood Pressure

[a] Effectiveness of intervention (net of control) in min / day on overall sedentary behaviour (SB) measured by self-report or device. When unreported, overall effects were loosely extrapolated from domain-specific effects (e.g., weekday and weekend reported separately) and exposure to the domain (e.g., 5 weekdays versus 2 weekend days/week). When only partial information was reported (e.g., workhours only) the extrapolation was based on the assumption of no effect outside of the reported domains. Percentages were converted to hours per day based on reported total amounts of time otherwise 8 hours working and 16 hours awake.

[a] For interim results reported in Balducci 2017, the corresponding value for effectiveness of intervention on sedentary behaviour is -33.0 min / day
